# MicroRNA profiling of CD3^+^CD56^+^ cytokine-induced killer cells

**DOI:** 10.1038/srep09571

**Published:** 2015-03-31

**Authors:** Wenju Wang, Ruhong Li, Mingyao Meng, Chuanyu Wei, Yanhua Xie, Yayong Zhang, Lihong Jiang, Ruiyi Dong, Chunhui Wang, Yiming Zhong, Fang Yang, Weiwei Tang, Xingfang Jin, Baohua Liu, Zongliu Hou

**Affiliations:** 1 Yan'an Affiliated Hospital of Kunming Medical University, Kunming 650051, Yunnan, People's Republic of China; 2 Yunnan Cell Biology and Clinical Translation Research Center, Kunming 650051, Yunnan, People's Republic of China; 3 Kunming Medical University, Kunming 650050, Yunnan, People's Republic of China; 4 First Affiliated Hospital of Kunming Medical University, Kunming 650031, Yunnan, People's Republic of China

## Abstract

Studies have proven that IL-2 and IL-15 showed contrasting roles during CIK cells preparation. By employing microarray, we analyzed miRNA expression profiles of PBMC, CIK_IL-2_ and CIK_IL-15_. Advanced bioinformatic analyses were performed to explore the key miRNAs which may regulate cell proliferation and anti-tumor activity of CIK. We identified 261 differentially expressed miRNAs (DEMs) between PBMC and CIK_IL-2_, and 249 DEMs between PBMC and CIK_IL-15_. MiR-143-3p/miR-145-5p was miRNA cluster which may positively regulate cell proliferation. In contrast, miR-340-5p/miR-340-3p cluster may negatively regulate cell proliferation via induction apoptosis, which may cause decreased cell proliferation capacity of CIK_IL-2._ MiRNA-target interaction analysis indicated that 10 co-downregulated miRNAs may synergistically turn on the expression of a pool of tumor cytotoxic genes in CIK cells. The DEMs between CIK_IL-2_ and CIK_IL-15_ may contribute to enhanced tumor cytotoxic capacity of CIK_IL-2_. Importantly, we found that repressed miR-193a-5p may regulate the expressions of inhibitory receptor KLRD1. The results of the validation assay have shown that KLRD1 were upregulated in CIK cells. Our findings have provided new insights into mechanisms of CIK cells production and tumor cytotoxic function, and shed light on their safety for clinical trial.

Amazing scientific advances have been translated into better ways to prevent, detect, diagnose and treat cancer during the past five years^1^. Nowadays, people are surviving longer after their cancer has been diagnosed due to these remarkable progress. Numerous therapeutics against cancer have shown large potential in clinical trials^1^. Notably, one group of strategies against cancer which are likely to revolutionize the treatment of certain cancer in the very near future are immunotherapies^1^. These therapeutics educate the patients' immune system to attack their cancer cells yielding both strong and durable response. Among these strategies, adoptive immunotherapy has shown great promise and encouraging efficacy in the tumor treatment with minimal adverse events^2^,^3^. Cytokine-induced killer (CIK) cells based immunotherapy is widely performed for clinical trials in China which is alternatives to conventional therapies^2^. CIK cells, a subset of T lymphocytes with a natural killer T cell phenotype, have been proven to be effective to most of tumors in vitro and in vivo^4^. CIK cells are generated from peripheral blood lymphocytes through time sequential stimulations of IFN-γ, monoclonal antibody against CD3 (OKT3) and IL-2. During this time period of CIK cells preparation, OKT3 provided mitogenic signals to T lymphocytes^5^. Priming with IFN-γ is to activate the monocytes through providing contact-dependent (CD58/LFA-3) and soluble (IL-12) crucial signals to promote generation of autophagy and antigen cross-presentation^6^. IL-2 is essential for T cell proliferation, survival and acquisition of cytolytic capacity in the following culture. At the end of expansion, a heterogeneous population of CD3^+^CD56^+^ CIK cells presenting potent cytotoxicity against a variety of tumor cells were obtained. However, the protocol for preparation of CIK cells can be differed for the purpose of enhancing the tumor cytotoxicity and CIK cells proliferation capacity^7^. It has been reported that the addition of IL-6 every 2–3 days during the preparation of CIK cells could inhibit the generation of Foxp3^+^ Treg cells and increase the proportion of CD3^+^CD56^+^ cells^8^. In our previous study, we have shown that CIK cells stimulated with combination of IL-2 and IL-15 exhibited enhanced proliferation capacity and cytotoxicity against lung cancer^9^. Interestingly, the results have indicated that CIK cells induced with combination of IL-2 and IL-15 could upregulate the expression levels of IFN-γ and TNF-α in mice models. In further investigation, we have found that CIK_IL-2_ showed greater tumor cytotoxicity than CIK_IL-15_, and CIK_IL-15_ exhibited enhanced proliferation capacity than CIK_IL-2_^10^. By advanced bioinformatic analysis of RNA-seq data from CIK_IL-2_ and CIK_IL-15_, results indicated that genes participating in Wnt signal pathway and focal adhesion were upregulated in CIK_IL-15_, and the expression levels of genes involved in cytokine-cytokine receptor interaction were increased in CIK_IL-2_^10^. Although the expression profiles of important genes in CIK_IL-2_ and CIK_IL-15_ have been well revealed, the regulation of these genes by IL-2 and IL-15 are still unknown.

MicroRNAs (miRNAs), a class of highly conserved ~20–22 nt long noncoding RNA, are essential molecules of post-transcriptional regulation of gene expression^11^. MiRNAs regulate gene expression negatively by targeting the 3′ untranslated region (3'UTR) or coding region of the mRNA, leading to either RNA degradation or inhibition of translation^12^. MiRNAs participated in many biological processes including cell proliferation, differentiation, apoptosis and tumorgenesis^13^. More recently, it was reported that miRNAs are involved in regulatory networks in immune system and regulation of development of immune cells^14^. However, the regulatory functions of miRNAs in CIK cells expansion and acquisition of cytotoxic capacity have not been reported yet.

In order to identify the roles of miRNAs in regulatory network of CIK cells generation, we performed miRNAs microarray analysis between PBMC (peripheral blood mononuclear cell) and CD3^+^CD56^+^ CIK cells, and investigated the changes in global miRNAs expression level. Advanced system biology strategies have been employed to comprehensively investigate the molecular mechanism of translational modulation of miRNAs during CIK cells expansion. Our finding will provide evidence to better understand the acquisition of tumor cytotoxicity and proliferation capacity of CIK cells.

## Results

### Dynamic miRNA profiles between PBMC and CIK cells

**Figure 1:**
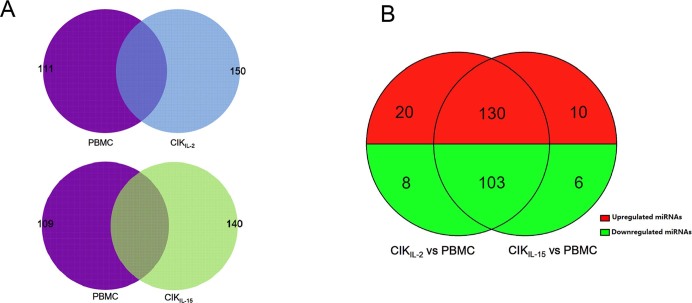
MiRNA expression profiles during CIK cells generation (A) Specific and shared differentially expressed miRNAs between PBMC and CIK cells (PBMC-CIK_IL-2_ and PBMC-CIK_IL-15_); (B) Comparative analysis of the differentially expressed miRNAs of CIK_IL-2_ and CIK_IL-15_.

We have prepared CIK_IL-2_ and CIK_IL-15_ from PBMCs of three healthy volunteers under identical conditions. Sequentially, PBMCs, CIK_IL-2_ and CIK_IL-15_ were sampled and preserved in liquid nitrogen for the following miRNA microarray analysis. The phenotype of CIK cells were determined by flow cytometry. The result showed that the average percentages of CD3^+^CD56^+^ cells were over 98% in both CIK_IL-2_ and CIK_IL-15_ (Figure S1 and Table S1). After confirmation of the portion of CD3^+^CD56^+^ cells, the proliferation capacity and tumor cytotoxicity of both CIK_IL-2_ and CIK_IL-15_ were measured by automatic absolute cell counting and CCK-8 based method respectively. The results of these two assays were previously described^10^. Then, miRNAs was isolated and purified from the preserved PBMC, CIK_IL-2_ and CIK_IL-15_. After quality assessment, the miRNAs were labeled and hybridized to Agilent V19.0 miRNA array. The array contained 2,006 human miRNAs, which allowed us to perform a deep investigation to CIK cells miRNA expression. After normalization of the raw data, we screened 261 differentially expressed miRNAs (DEMs) between PBMC and CIK_IL-2_, and 249 DEMs between PBMC and CIK_IL-15_ by the following criteria: fold change (FC) > 2 or FC < 0.5, P value < 0.05, all flag signals of 3 replicate were all same (Table S2 and S3). Of these DEMs between PBMC and CIK_IL-2_, 111 and 150 miRNAs were downregulated and upregulated respectively (Figure 1A). There were 109 downregulated and 140 upregulated miRNAs between PBMC and CIK_IL-15_ (Figure 1A). However, no significant DEM was identified between CIK_IL-2_ and CIK_IL-15_ by the screening criteria referred above. By further comparison of the miRNA expression patterns of CIK_IL-2_ and CIK_IL-15_ against PBMC, we found that 130 miRNAs were co-upregulated, accounting for 86.66% and 92.85% of total upregulated miRNAs in CIK_IL-2_ and CIK_IL-15_ respectively. In addition, there were 103 miRNAs co-downregulating in both of the two CIK cells, accounting for 92.79% and 94.49% of total downregulated miRNAs in CIK_IL-2_ and CIK_IL-15_ respectively (Figure 1B).

### Differentially expressed miRNA chromosome clustering

**Figure 2:**
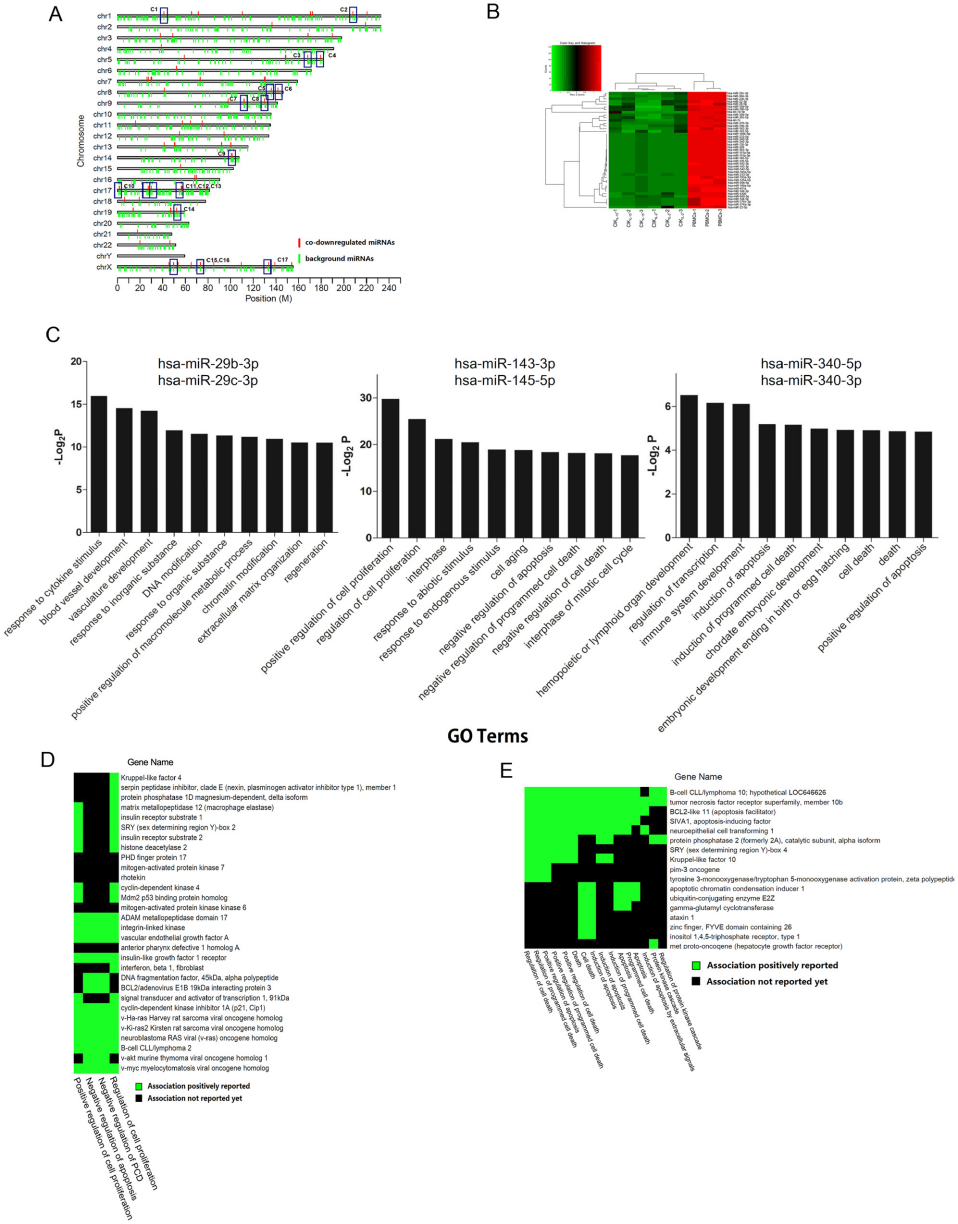
Chromosome clustering and gene ontology analysis (A) Chromosome clustering analysis of co-downregulated miRNAs. Red lines and green lines represent co-downregulated miRNAs and background miRNAs on each chromosome respectively; (B) Heatmap and hierarchical clustering of differentially expressed miRNAs which are chosen for chromosome clustering and miRNA-targets interaction network analysis. (C) Significant gene ontology analysis of 3 co-downregulated miRNAs clusters including miR-29b-3p/miR-29c-3p, miR-143-3p/miR-145-5p and miR-340-5p/miR-340-3p. P value < 0.05 for all significant GO terms. (D) 2-D view analyses establish associations between GO terms participating in promoting cell proliferation and genes regulated by miR-143-3p/miR-145-5p cluster. (E) 2-D view analyses between induction of apoptosis (GO term) and genes regulated by miR-340-5p/miR-340-3p.

Interestingly, evidences have supported that CIK_IL-2_ and CIK_IL-15_ shared over 90% of downregulated miRNAs. Furthermore, target genes may be upregulated in response to downregulation of corresponding miRNA. Therefore, co-downregulated miRNAs were chosen for further analysis. In order to identify their function during CIK preparation, we aligned the co-downregulated miRNAs to the chromosomes they located based on the chromosome coordinates of each miRNA (Figure 2A). We assumed that miRNAs which closed to each other may have the same biological function^15^,^16^. Among co-downregulated miRNAs, we have found 17 miRNA clusters in which the distance of miRNAs are no longer than 5000 bp (Figure 2A, Table 1). The heatmap and hierarchical analysis of 17 DEMs clusters were shown in Figure 2B. There were 3 miRNA clusters in co-upregulated miRNAs. No miRNA cluster were observed in CIK_IL-2_ or CIK_IL-15_ specific DEMs.

### Predicted target gene ontology and pathway analysis

**Figure 3:**
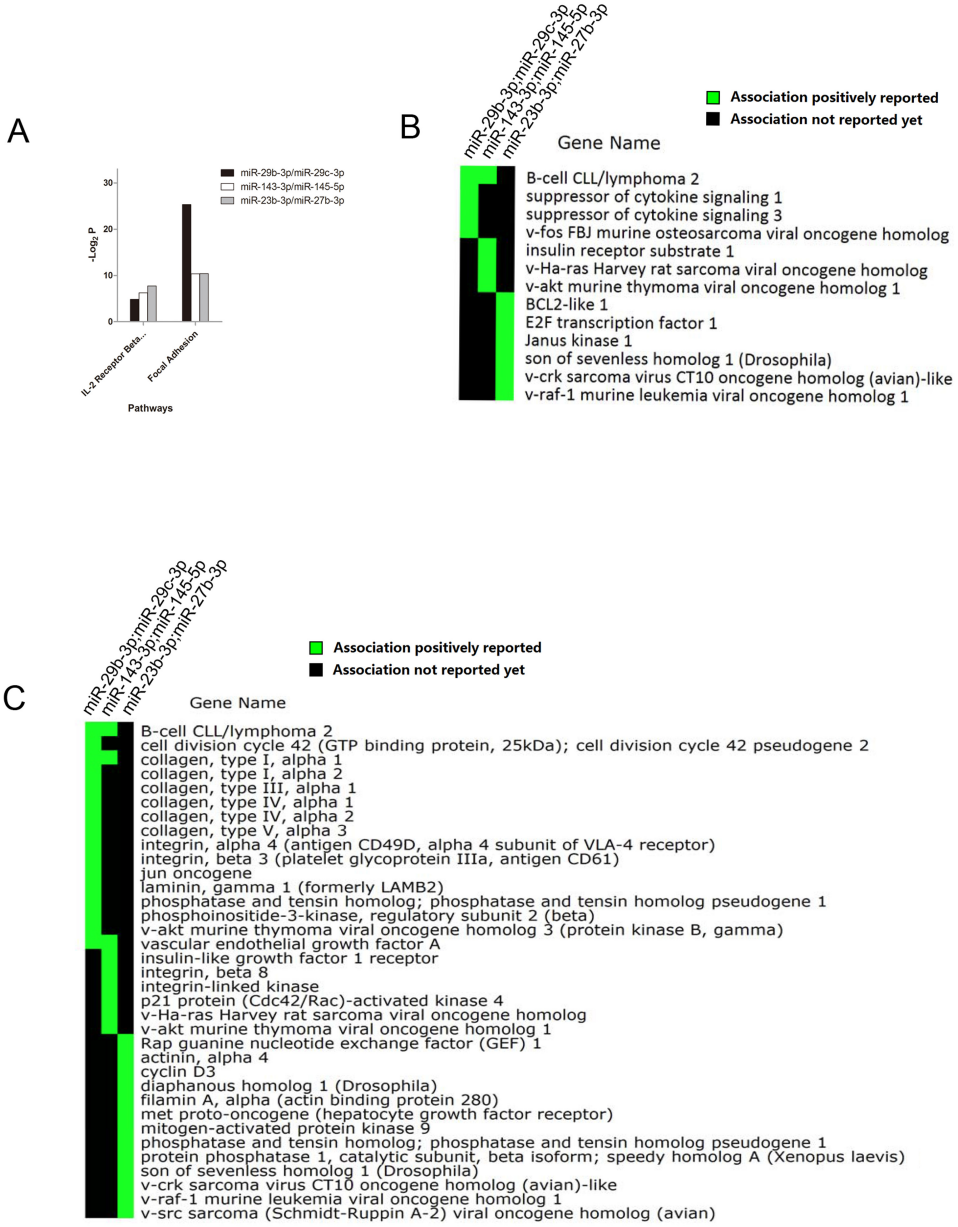
Pathway analysis of co-downregulated miRNA clusters based on KEGG and Biocarta (A) Significant pathway analysis of miR-29b-3p/miR-29c-5p, miR-143-3p/miR-145-5p and miR-23b-3p/miR-27b-3p. P value < 0.05 for all significant pathways.; (B) 2-D view analysis of IL-2 receptor beta chain in T cell activation pathway. Associations between genes involved in this pathway and their regulatory miRNA clusters were shown; (C) 2-D view analysis of focal adhesion pathway. Green panes represent associations have already reported. Black panes represent associations have not been reported yet.

To further characterize the function of the miRNA we identified, the target genes of each miRNA cluster were predicted based on miRTarBase Release 4.5. The target genes of each miRNA in miRTarBase were validated by Reporter assay, Western Blot, Microarray or pSILAC. Next, we performed gene ontological analysis to screen co-downregulated miRNA clusters whose target genes may be involved in cell proliferation and tumor cytotoxicity of CIK cells by employing annotation tool of DAVID bioinformation database. By analyzing the significant GO terms, we found that response to cytokine stimulus and positive regulation of cell proliferation were the most significant GO terms of the target genes regulated by miR-29b-3p/miR-29c-3p (C2) and miR-143-3p/miR-145-5p (C3) respectively (Figure 2C). Importantly, induction of apoptosis was significant among target genes of miR-340-5p/miR-340-3p (C4) (Figure 2C). We focused on cell proliferation and induction of apoptosis which are two key biological functions of CIK cells. Functional classification analysis in 2-D view was performed in order to visualize the associations between target genes of C3 and C4 clusters and GO terms including cell proliferation and induction of apoptosis (Figure 2D, Figure 2E). With respect to miR-143-3p/miR-145-5p cluster, HRAS, KRAS and NRAS which are the most common members of Ras subfamily were found to be strongly correlated with cell proliferation of CIK cells. By 2-D view analysis, two proto-oncogenes including Bcl2 and c-Myc which are involved in promoting cell proliferation were identified to be regulated by miR-143-3p/miR-145-5p cluster (Figure 2D). Among the genes regulated by miR-340-5p/miR-340-3p, TNFRSF10B was shown to participate in cell death (Figure 2E). To further explore the influence of co-downregulated miRNA cluster on function of CIK cells, we performed pathway analysis based on KEGG and Biocarta databases by using Fisher exact test. The results indicated that target genes of miR-29b-3p/miR-29c-3p, miR-143-3p/miR-145-5p and miR-23b-3p/miR-27b-3p clusters were all involved in IL-2 receptor beta chain in T cell activation and focal adhesion. The two pathways were significant in the target genes of each miRNA cluster (Figure 3A). By 2-D view analysis, the genes regulated by the 3 miRNA clusters were mainly involved in cell proliferation signaling transduction pulsed by IL-2 including kinases (JAK1, RAF1, CRKL and AKT1), signaling transducers (SOS1, HRAS, SOCS1, SOCS3 and IRS1), DNA binding proteins (FOS and E2F1) and apoptosis suppressors (BCL2 and BCL2L1) (Figure 3B). Among genes involved in focal adhesion, we found that components of extracellular matrix and their receptors were regulated by miR-29b-3p/miR-29c-3p including collagen, laminin and integrin (Figure 3C). Additionally, target genes regulated by miR-143-3p/miR-145-5p and miR-23b-3p/miR-27b-3p clusters were responsible for signal transduction which may be mediated by integrin.

### MiRNA-Target Interaction (MTI) network in tumor cytotoxicity pathway

**Figure 4:**
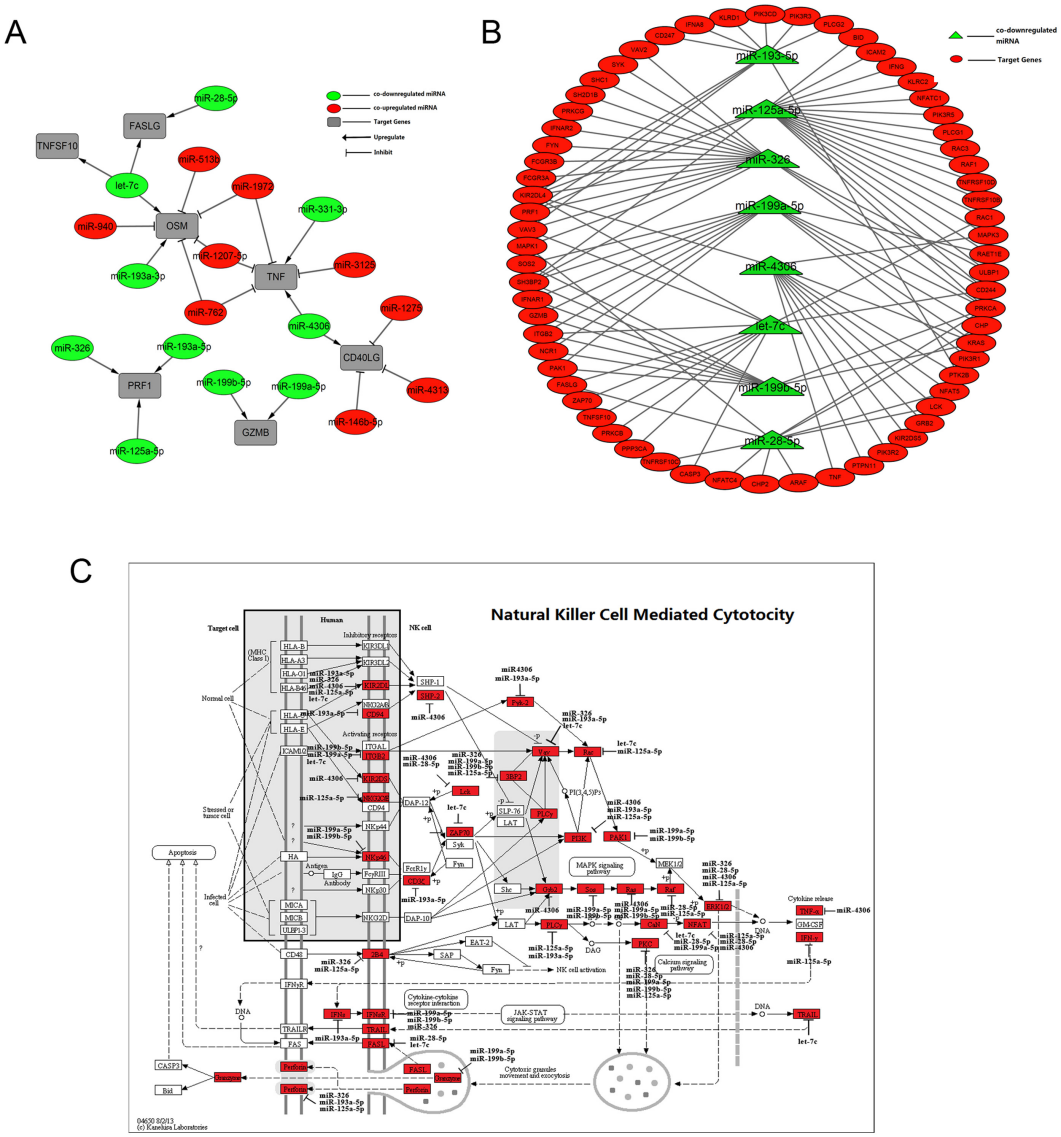
MiRNA-target interactions network analysis of tumor cytotoxicity (A) Regulatory network of tumor cytotoxic genes and shared differentially expressed miRNAs; (B) Interaction network between tumor cytotoxicity promoting miRNAs and components of natural killer cells mediated cytotoxicity pathway; (C) Potential impact of tumor cytotoxicity promoting miRNAs in natural killer cells mediated cytotoxicity pathway. Components of signaling pathway which were regulated by miRNAs we identified were marked in red, and corresponding regulatory miRNAs were listed beside.

Alternatively, we have employed miRTar database to identify miRNA-target interactions in cytotoxicity pathway. We inputted co-downregulated miRNAs and selected natural killer cell mediated cytotoxicity and cytokine-cytokine receptor interaction as our target pathways which were derived from KEGG database. We built the miRNA-target interaction network based on the anti-tumor factors expressed on cytotoxic lymphocytes (Figure 4A). The network analysis indicated that 10 distinct co-downregulated miRNA were found targeting cytotoxic genes, and the hierarchical analysis of their expression profiles was shown in figure 2B. The results indicated that let-7c was a key miRNA which regulated 3 tumor toxic molecules including Fas ligand (FasL), TNFSF10 (Trail) and OSM (Oncostatin M). Among the cytotoxic genes, PRF1, GZMB, TNFSF10 and FasL were shown to be targeted only by co-downregulated miRNAs. Other anti-tumor genes including OSM, TNF-α and CD40LG were regulated by miRNAs of both downregulated and upregulated. In addition, we performed miRNAs-pathway interaction analysis to establish the linkage between these 10 miRNA and gene pool participating in natural killer cell mediated cytotoxicity pathway. By building the miRNA-pathway network, the results showed that 8 co-downregulated miRNAs were involved in regulation of natural killer cell mediated cytotoxicity pathway, and the correlations between them were shown in figure 4B. In the gene group regulated by these 8 miRNAs, we found that receptors, signal transduction components and tumor cytotoxicity factors were all included (Figure 4C). Among receptors expressed on NK cells, inhibitory receptors (KIR2DL4; CD94) and activating receptors (ITGB2; KIR2DS5; NKG2C/E; NKp46) were identified which may work as sensors to discriminate normal cells and tumor cells.

### Validation of representative miRNAs and mRNAs

**Figure 5:**
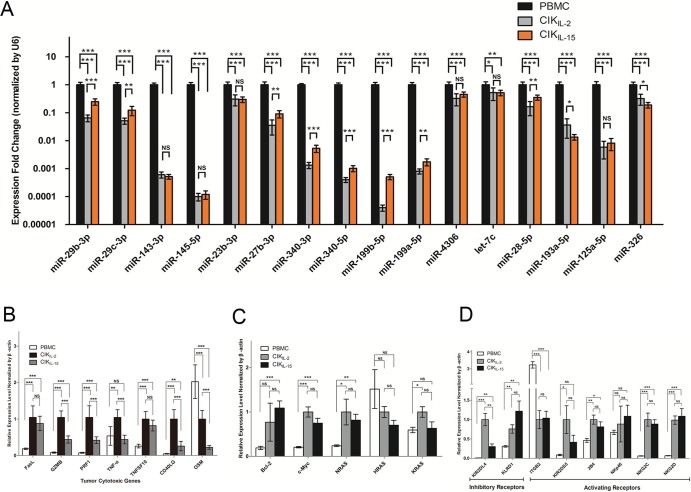
qRT-PCR validation of relative expression levels of representative co-downregulated miRNAs and their targets. (Data are plotted as mean ± SEM *P < 0.05; **P < 0.01; ***P < 0.0001; NS no significance) (A) Confirmation of the differentially expressed miRNAs by real-time PCR analysis. The miRNA expression was normalized with U6. The scale of Y-axis was presented in Log_2_Scale; (B) Validation of differentially expressed tumor cytotoxic genes between PBMC and CIK cells; (C) qRT-PCR analysis of the expression profiles of proto-oncogenes participating in cell proliferation of CIK cells; (D) Confirmation of differentially expressed inhibitory and activating receptors in PBMC, CIK_IL-2_ and CIK_IL-15_. NKG2D, which has been reported to be upregulated in CIK cells was taken as positive control. The expressions of protein coding genes were normalized with β-actin.

**Figure 6:**
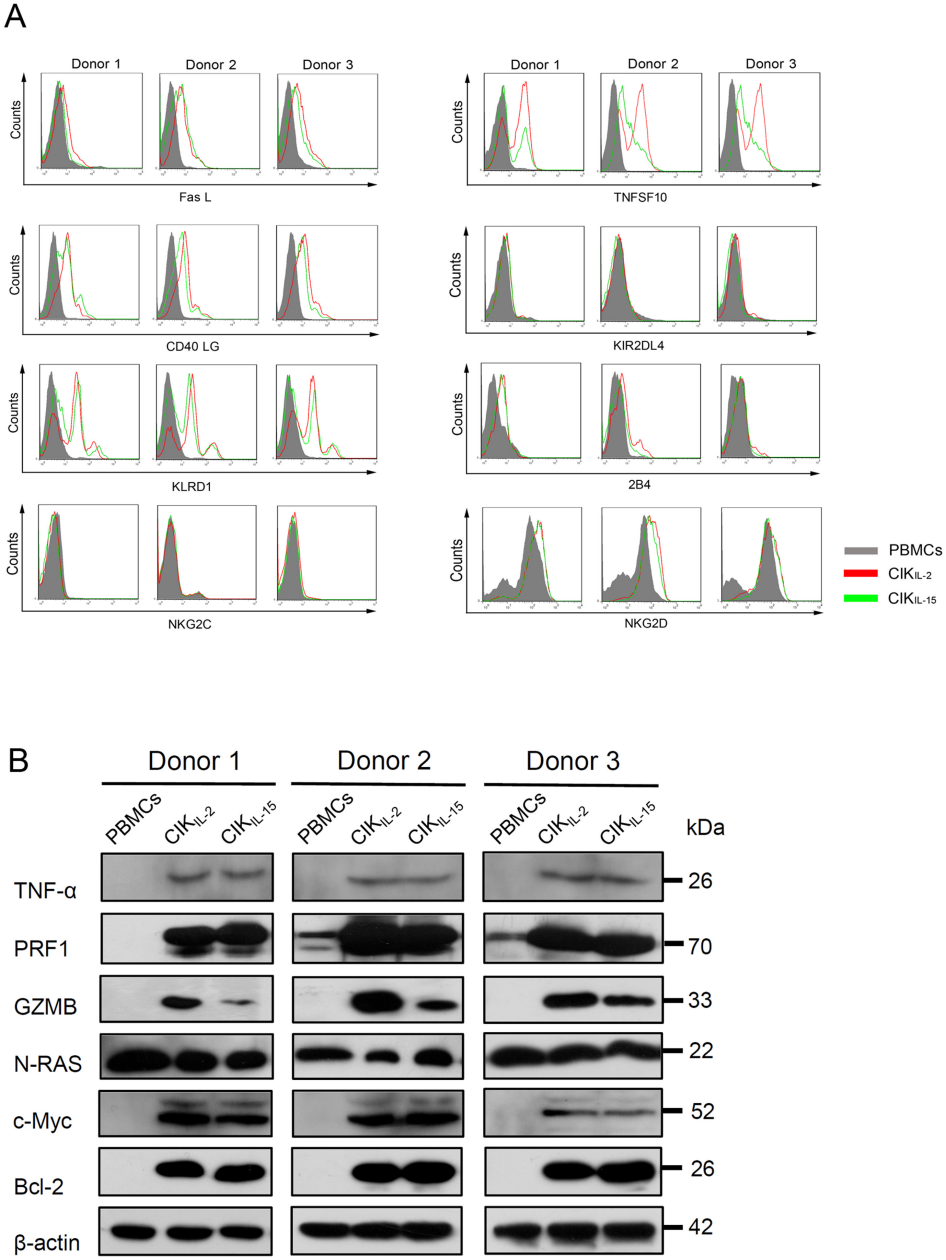
Flow cytometry and Western blot analysis of target genes. (A) Flow cytometry analysis of the cytotoxic genes, inhibitory and activating receptors which are expressed on cell surface. Results were obtained in three independent samples. (B) PBMCs, CIK_IL-2_ and CIK_IL-15_ were lysed followed by cell total protein determination. The total protein amount were normalized for western blot analysis. Three independent samples were included.

Next, we examined the expression profiles of co-downregulated miRNAs referred above by employing qRT-PCR across these 3 types of cells. Consistent with miRNA array data, the results have shown that all miRNAs we referred above were significantly downregulated in both CIK_IL-2_ and CIK_IL-15_ compared to PBMC (Figure 5A). Interestingly, among these 16 miRNAs, 10 miRNAs differentially expressed between CIK_IL-2_ and CIK_IL-15_, which have not been identified by microarray analysis (Figure 5A). Moreover, we validated the expression levels of cytotoxic genes in CIK cells in order to demonstrate the regulatory relationship between miRNAs of interest and their targets we suggested above. Except for OSM, the expression of all anti-tumor genes including TNF-α, PRF1, GZMB, FasL, TNFSF10 and CD40LG were significantly upregulated in CIK cells compared to PBMC, which were negatively correlated with expression profiles of their potential regulators (Figure 5B, Figure 6A and Figure 6B). Among these genes, the expression of GZMB and TNFSF10 were significantly higher in CIK_IL-2_ than CIK_IL-15_ in protein level (Figure 6A and Figure 6B). To further explore the cell proliferation mechanism of CIK cells, the expression of important genes regulated by miR-143-3p/miR-145-5p cluster were profiled. The results indicated that the expressions of c-Myc and Bcl-2 were significantly increased in CIK_IL-2_ and CIK_IL-15_ (Figure 5C and Figure 6B). Although NRAS mRNA was found to be significantly upregulated in CIK cells, no significant difference was observed in protein level (Figure 5C and Figure 6B). Importantly, we have examined the expression of inhibitory and activating receptors to evaluate the recognition specificity of CIK cells. NKG2D, which has been reported to be upregulated in CIK cells was taken as positive control in validation assay. By validation of both mRNA and protein level, the results showed only KLRD1 (CD94) were significantly upregulated in CIK cells (Figure 5D and Figure 6A).

## Discussion

Nowadays, immunotherapies which have made remarkable progress are revolutionizing the treatment of cancers. However, immunotherapy development is a challenging work which need large amount of studies to prove its effectiveness and safety. CIK is one of adoptive immunotherapy approaches which have exhibited potent cytolytic activities against tumor cells with minimal adverse effects. The original work of CIK study was reported by Schmidt-Wolf from Stanford^4^. Clinical trials of CIK based immunotherapies were widely performed in China, however, few studies were observed which focused on molecular mechanism of tumor toxic function. Herein, we performed microarray analysis to identify the miRNA expression profiles of CD3^+^CD56^+^CIK cells for the first time, and elucidate their proliferation and cytolytic mechanisms on post-transcriptional regulation level. The efficiency of CIK based immunotherapies were determined by cell proliferation and cytotoxicity capacities against tumor^5^. To improve the effectiveness of CIK cells, cytokines including IL-1, IL-17, IL-12 and IL-15 were used instead of IL-2 or in combination with IL-2^7^. IL-2 and IL-15 have similar biological function in vitro, and they shared receptor signaling components (IL-2/15Rβγ_c_) with each other^17^,^18^. By pathway analysis, we found co-downregulated miRNA clusters including miR-29b-3p/miR-29c-3p, miR-143-3p/miR-145-5p and miR-23b-3p/miR-27b-3p were participated in IL-2 receptor beta chain in T cell activation (Figure 3A and 3B). However, contrasting roles of IL-2 and IL-15 were observed in lymphocytes based immunotherapy^19^. It was reported that CIK cells which were generated in combination with IL-2 and IL-15 showed greater cytotoxicity against lung cancer than CIK cells prepared with IL-2 alone^9^. Moreover, our previous comparative analysis indicated that CIK_IL-15_ showed enhanced cell proliferation capacity than CIK_IL-2_, whereas, CIK_IL-2_ showed greater tumor cytotoxic activity against tumor than CIK_IL-15_ in vivo^10^. Interestingly, advanced bioinformatic analysis and validation assays provided evidences to illuminate the potential mechanism of differential cell proliferation capacity and anti-tumor activity of CIK_IL-2_ and CIK_IL-15_.

We have identified 130 co-downregulated and 103 co-upregulated miRNAs through microarray analysis. By chromosome clustering and miRNA-target interaction analysis, we have focused on 16 miRNAs for further qRT-PCR validation assay. We have noticed that it was important to determine the abundance of miRNAs of interested because that very minimally expressed miRNA which showed dramatically change may be unimportant during biological process. Studies based on two distinct next-generation platforms have suggested that approximately 60 mature miRNA sequences comprised over 97% of miRNA sequences in mouse natural killer cells (NK cells)^20^,^21^. The results have shown that miR-21 was ranked as number 1 miRNA which accounted for nearly 31% of the miRNA sequence in NK cells^21^. Surprisingly, we have found that miR-29b, miR-27b, let-7c and miR-28 we included in this study were listed within the top 60 miRNAs which accounted for over 97% miRNA sequence in NK cells.

By chromosome clustering and GO analysis, we found that miR-143-3p/miR-145-5p were a miRNA cluster which may positively regulated the cell proliferation of CIK cells. Importantly, proto-oncogene including Ras family, Bcl-2 and c-Myc were predicted targets of this miRNA cluster. However, we found that only Bcl-2 and c-Myc were significantly upregulated during CIK generation (Figure 5C and Figure 6B). These two anti-apoptotic genes may be involved in cell proliferation during CIK preparation^22^. On the other hand, target genes of miRNA cluster containing miR-340-5p and miR-340-3p was identified to potentially participate in induction of apoptosis (Figure 2C and 2E). The results of qRT-PCR showed that the expression level of miR-340-5p and miR-340-3p were significantly higher in CIK_IL-15_ than CIK_IL-2_, which indicated that a pool of apoptosis promoting genes may be upregulated in CIK_IL-2_ compared to CIK_IL-15_. Furthermore, IL-15 is an anti-apoptotic cytokine which inhibits IL-2 mediated activation-induced cell death (AICD) of T cell and stimulates survival of memory T cell, whereas, IL-2 induces AICD and eliminates self-reactive T cell to maintain peripheral tolerance through upregulating of FasL and TNFSF10^19^,^23^. Interestingly, we have found that the expression of FasL and TNFSF10 were upregulated in both CIK_IL-2_ and CIK_IL-15_. However, the expression of TNFSF10 was higher in CIK_IL-2_ than CIK_IL-15_ (Figure 6B). Collectively, these evidences may explain our observation that CIK_IL-15_ showed greater proliferation potential than CIK_IL-2_ as previously described.

We have built the regulatory network among tumor toxic genes and differentially expressed miRNA which governed their expression. It showed that GZMB, which is a key anti-tumor molecule in vivo, was regulated mainly by miR-199a-5p and miR-199b-5p. Data from qRT-PCR and Western blot suggested that the expression level of GZMB was significantly increased in CIK cells. Importantly the results have implicated that the expression of GZMB was upregulated in CIK_IL-2_ compared to CIK_IL-15_, which negatively correlated with the expression profiles of miR-199a-5p and miR199b-5p and suggested the enhance tumor cytotoxicity of CIK_IL-2_. PRF1, which is known as pore-forming protein and induces apoptosis in synergy with GZMB was upregulated in both CIK_IL-2_ and CIK_IL-15_^24^,^25^. Except for TNF-α and TNFSF10 which have been reported as anti-tumor molecules of CIK, we have identified CD40LG as tumor toxic effector in CIK cells by analyzing interactions between co-downregulated miRNAs and cytokine-cytokine receptor interaction pathway^26^,^27^,^28^. The expression of CD40 LG was upregulated in CIK cells. Compared to PBMC, OSM was significantly downregulated in both CIK_IL-2_ and CIK_IL-15_. Studies have reported that OSM which was initially found to inhibit proliferation of several tumors appeared to promote growth of malignant cells now^29^,^30^,^31^. Therefore, evidences obtained from miRNA-Target network and validation assays have suggested the potential cytotoxicity mechanism of CIK cells. The higher expression of TNFSF10 and GZMB in CIK_IL-2_ may account for the greater tumor cytotoxic efficiency of CIK_IL-2_ than CIK_IL-15_.

Additionally, the function of natural killer cell is determined by the balance between signals triggered by activating and inhibitory receptors. CD94-NKG2A are important inhibitory receptor systems in most species, and they transduce inhibitory signal through SHP-1 and SHP-2^32^,^33^,^34^. By co-downregulated miRNAs-receptor interaction analysis, we found that KLRD1 (CD94) were significantly upregulated in CIK cells. KLRD1 is a peptide-selective receptor on NK cell, which binds HLA-E-peptide complex and provides inhibitory signal in absence of its signaling partner NKG2A^35^. This results suggested that increased expression of CD94 in CIK cell may protect HLA-E positive cell from lysis^36^.

In conclusion, we have performed microarray analysis to investigate the dynamic miRNA expression profiles during CIK cell preparation for the first time. By advanced bioinformatic analysis, the results indicated that miR-143-3p/miR-145-5p cluster may positively regulated cell proliferation through upregulating a group of proto-oncogenes. In contrast, miR-340-5p/miR-340-3p cluster may negatively regulated cell proliferation via induction apoptosis, which may cause decreased cell proliferation potential of CIK_IL-2._ MiRNA-target interaction analysis revealed that 10 co-downregulated miRNAs may synergistically promote the expression of a pool of tumor cytotoxic genes in CIK cells. Importantly, upregulation of inhibitory receptors (KLRD1) in CIK cells implicated the possibility that activation of CIK cells through activating receptors (NKG2D) could be negatively regulated by KLRD1. These evidences suggested that the presences of both activating and inhibitory receptors on CIK cells may contribute to their safety for clinical trial.

## Methods

### Antibodies and Cytokines

The antibodies for CIK cells phenotype assay were purchased from BD Biosciences. The antibodies used for detecting cell surface receptors and cytotoxic factors were obtained from BioLegend, Inc. and R&D System. For Western blot, antibodies were purchased from EMD Millipore and Santa Cruz Biotechnology, Inc. Cytokines for CIK cells preparation including OKT3, IFN-γ, IL-2 and IL-15 were from Miltenyi Biotec. Methods involving human peripheral blood in this studies were reviewed and approved by Bioethics Committee of Yan'an Affiliated Hospital of Kunming Medical University. The methods were carried out in accordance with the approved guidelines. Written informed consents have been given from all volunteers participated in this study.

### Generation of CIK cells

The Bioethics Committee of Yan'an Affiliated Hospital of Kunming Medical University has approved the investigation protocols to draw blood from healthy volunteers after written informed consent for the purposes of CIK cells preparation against tumor and microarray analysis. The standard protocol of CIK generation was described previously^9^. Briefly, PBMCs were isolated by standard Ficoll separation and cultured in RPMI 1640 growth medium at a density of 5 × 10^6^ cells/mL. The RPMI 1640 growth medium for CIK cell contained 10% FBS, 2% L-glutamine and antibiotics. The generation of CIK cells was primed by adding 1000 U/mL IFN-γ on day 0 and 100 ng/mL anti-CD3 antibody and 500 U/mL IL-2 or 10 ng/mL IL-15 within the following 15 days of culture. The CIK cells were propagated every 5 days with RPMI 1640 growth medium supplemented with anti-CD3 antibody and IL-2 or IL-15 respectively. The CIK cells were expanded for 15 days.

### RNA isolation and labeling

Total RNA was extracted and purified using mirVana™ miRNA Isolation Kit (AM1560, Ambion, Austin, TX, US) following the manufacturer's instructions. The quality was assessed by an Agilent Bioanalyzer 2100 (Agilent technologies, Santa Clara, CA, US). MiRNA in total RNA was labeled by miRNA Complete Labeling and Hyb Kit (5190-0456, Agilent technologies, Santa Clara, CA, US) following the manufacturer's instructions.

### Microarray hybridization

Labeled RNA samples were further detected by the miRNA array by using Agilent's human miRNA microarray, version 19.0. Each slide was hybridized with 100 ng Cy3-labeled RNA using miRNA Complete Labeling and Hyb Kit in hybridization Oven (G2545A, Agilent technologies, Santa Clara, CA, US) at 55°C, 20 rpm for 20 hours according to the manufacturer's instructions. After hybridization, slides were washed in staining dishes (121, Thermo Shandon, Waltham, MA, US) with Gene Expression Wash Buffer Kit (5188–5327, Agilent technologies, Santa Clara, CA, US).

### Data acquisition and identification of differentially expressed miRNAs

Slides were scanned by Agilent Microarray Scanner (G2565BA, Agilent technologies, Santa Clara, CA, US) and Feature Extraction software 10.7 (Agilent technologies, Santa Clara, CA, US) with default settings. Raw data were normalized by Quantile algorithm, Gene Spring Software 11.0 (Agilent technologies, Santa Clara, CA, US). Differentially expressed miRNAs were identified by using unpaired Student's t test with P values cutoff by 0.05 and fold change more than 2.0 or less than 0.5.

### Target genes prediction, Gene ontology and pathway analysis

MicroRNA target gene prediction in gene ontology analysis was performed by miRTarBase Release 4.5 which is a public platform providing known experimentally validated miRNA targets^37^. GO analysis was applied to analyze the main function of the targets of differentially expressed miRNAs according to the Gene Ontology which is the key functional classification of NCBI^38^,^39^. GO analysis of target genes were performed by employing DAVID gene annotation tool^40^,^41^. Statistical analysis of GO terms was done by Fisher's exact test and *x*^2^ test, and the false discovery rate (FDR) was calculated to correct the P-value,the smaller the FDR, the small the error in judging the p-value. The significant GO terms were defined as P value < 0.05 and FDR < 0.05. Likewise, pathway analysis was used to find out the significant pathway of target genes of differentially expressed miRNAs according to KEGG and Biocarta^42^. Still, we turned to the Fisher's exact test and *x*^2^ test to select the significant pathway, and the threshold of significance was defined by P-value and FDR. The significant pathway was identified by P value < 0.05 and FDR < 0.05. We visualized the associations between target genes and miRNAs\pathways by using functional classification 2-D view analysis module of DAVID annotation tool. MiRNA-target interaction was analyzed by miRTar web server of human (http://mirtar.mbc.nctu.edu.tw/human/). We picked the miRNA-target interactions in biological pathways of interest and used Cytoscape for graphical representations.

### Quantitative reverse transcription PCR

The qRT-PCR was performed on the CFX96 Touch™ (BIORAD, USA). The first strand of cDNA was synthesized with adjusted concentration of RNA, and corresponding genes were amplified by employing EVA Green Supermix (BIORAD, USA). All the primers used for qRT-PCR were obtained from GeneCopoeia (USA).

### Flow cytometry

The cells were collected by centrifugation at speed of 2000 rpm. The cell pellets were suspended with blocking buffer. After washing with blocking buffer, the cells were stained with corresponding mAbs for 30 min at room temperature. The monoclonal antibodies (MAb) used were either conjugated with fluorescein isothiocyanate (FITC), phycoerythrin (PE) or phycoerythrin-cyanin 5 (PerCP). Cell surface markers of FasL, NKG2D and TNFSF10 were labeled with PE conjugated mAb. CD40LG, CD94, KIR2DS5 and KIR2DL4 were stained by FITC conjugated mAb. 2B4 and NKG2C were stained with PerCP labeled mAbs against corresponding markers. After staining, the cells were washed twice before FACS analysis.

### Western Blot

PBMCs, CIK_IL-2_ and CIK_IL-15_ were treated with cell lysis buffer, and the concentration of total proteins extracted were measured by Lowry based method. The samples were analyzed by 12% SDS-PAGE gel loaded with equal amounts of protein. The proteins were electransferred to PVDF membrane at 40 V for 100 min. Next, the membrane was incubating with 5% skimmed milk in PBST for blocking overnight. The primary antibodies against TNF-α, PRF1, Bcl-2, GZMB, c-Myc, N-RAS and β-actin were added and incubated at room temperature for 4 hours. The HRP conjugated secondary antibodies were added after three time PBST washing. After incubating, the membranes were washed thoroughly with PBST for 4 times, and then the bands were visualized by enhanced chemiluminescence kit (Millipore, USA).

## Supplementary Material

Supplementary Information supplementary information

## Figures and Tables

**Table 1:** Chromosome clusters of co-downregulated miRNAs between PBMC and CD3^+^CD56^+^ CIK cells

Systemic Name	miRBase Accession No	Chromosome	Start	End
C1				
hsa-miR-30c-5p	MIMAT0000244	Chr1	41222977	41222994
hsa-miR-30e-5p	MIMAT0000692	Chr1	41220048	41220064
hsa-miR-30e-3p	MIMAT0000693	Chr1	41220089	41220106
C2				
hsa-miR-29b-3p	MIMAT0000100	Chr1	207975861	207975841
hsa-miR-29c-3p	MIMAT0000681	Chr1	207975271	207975250
C3				
hsa-miR-143-3p	MIMAT0000435	Chr5	148808547	148808561
hsa-miR-145-5p	MIMAT0000437	Chr5	148810229	148810246
C4				
hsa-miR-340-5p	MIMAT0004692	Chr5	179442339	179442321
hsa-miR-340-3p	MIMAT0000750	Chr5	179442381	179442361
C5				
hsa-miR-30b-5p	MIMAT0000420	Chr8	135812800	135812783
hsa-miR-30d-5p	MIMAT0000245	Chr8	135817145	135817132
C6				
hsa-miR-151a-5p	MIMAT0004697	Chr8	141742693	141742677
hsa-miR-151a-3p	MIMAT0000757	Chr8	141742729	141742713
C7				
hsa-miR-23b-3p	MIMAT0000418	Chr9	97847551	97847567
hsa-miR-27b-3p	MIMAT0000419	Chr9	97847790	97847807
C8				
hsa-miR-126-3p	MIMAT0000445	Chr9	139565109	139565126
hsa-miR-126-5p	MIMAT0000444	Chr9	139565068	139565088
C9				
hsa-miR-376a-3p	MIMAT0000729	Chr14	101506457	101506475
hsa-miR-376c-3p	MIMAT0000720	Chr14	101506071	101506089
C10				
hsa-miR-22-5p	MIMAT0004495	Chr17	1617232	1617215
hsa-miR-22-3p	MIMAT0000077	Chr17	1617270	1617252
C11				
hsa-miR-451a	MIMAT0001631	Chr17	27188424	27188403
hsa-miR-144-5p	MIMAT0004600	Chr17	27188586	27188565
hsa-miR-144-3p	MIMAT0000436	Chr17	27188621	27188602
C12				
hsa-miR-193a-5p	MIMAT0004614	Chr17	29887045	29887056
hsa-miR-193a-3p	MIMAT0000459	Chr17	29887075	29887090
C13				
hsa-miR-142-5p	MIMAT0000433	Chr17	56408628	56408611
hsa-miR-142-3p	MIMAT0000434	Chr17	56408666	56408645
C14				
hsa-miR-99b-5p	MIMAT0000689	Chr19	52195877	52195892
hsa-let-7e-5p	MIMAT0000066	Chr19	52196049	52196067
hsa-miR-125a-5p	MIMAT0000443	Chr19	52196527	52196544
C15				
hsa-miR-362-3p	MIMAT0004683	ChrX	49773616	49773634
hsa-miR-660-5p	MIMAT0003338	ChrX	49777867	49777885
C16				
hsa-miR-223-5p	MIMAT0004570	ChrX	65238737	65238758
hsa-miR-223-3p	MIMAT0000280	ChrX	65238780	65238800
C17				
hsa-miR-542-5p	MIMAT0003340	ChrX	133675408	133675390
hsa-miR-542-3p	MIMAT0003389	ChrX	133675444	133675424
