# Supplementary material for: MicroRNA profiling of CD3+CD56+ cytokine-induced killer cells
Source: Sci Rep. 2015 Mar 31;5:9571. doi: 10.1038/srep09571 (PMC5380330; doi:10.1038/srep09571)
Supplement: Supplementary Information [file srep09571-s1.pdf]

MicroRNA profiling of $CD3^{+}CD56^{+}$ cytokine-induced killer cells

Wenju Wang [1,2*], Ruhong Li [1,2], Mingyao Meng [1,2], Chuanyu Wei [1,2], Yanhua Xie [1,2], Yayong Zhang [1], Lihong Jiang [1], Ruiyi Dong [1,2], Chunhui Wang [1,2], Yiming Zhong [1,2], Fang Yang [2,3], Weiwei Tang [1,2], Xingfang Jin [1,2], Baohua Liu [3], Zongliu Hou [1,2*]

[1]*Yan'an Affiliated Hospital of Kunming Medical University, Kunming 650051, Yunnan, People's Republic of China*

[2]*Yunnan Cell Biology and Clinical Translation Research Center, Kunming 650051, Yunnan, People's Republic of China*

[3]*Kunming Medical University, Kunming 650050, Yunnan, People's Republic of China*

[4]*First Affiliated Hospital of Kunming Medical University, Kunming 650031, Yunnan, People's Republic of China*

*Corresponding Author

Wenju Wang Ph D

Department of Central Laboratory, Yan'an Affiliated Hospital of Kunming Medical University. Address: No. 245 East of Renmin Road, Kunming, Yunnan, People's Republic of China. Postal code: 650051; Tel: +86-0871-63211157, E-mail: wangwenju_vip@hotmail.com

Zongliu Hou MD

Department of Central Laboratory, Yan'an Affiliated Hospital of Kunming Medical University Address: No. 245 East of Renmin Road, Kunming, Yunnan, People's Republic of China. Postal code: 650051; Tel: +86-0871-63211157, E-mail: hzl579@163.com

Supplementary Figure

Figure S1

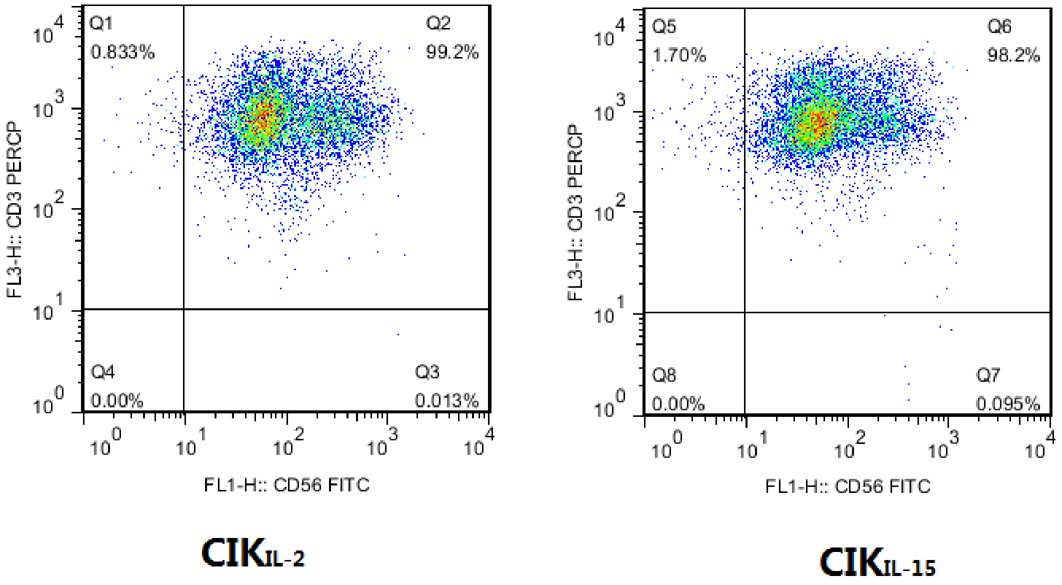

Figure S1 Measurement of the purity of $CD3^+CD56^+$ CIK cells

Supplementary Table

Table S1

The purity of $CD3^+CD56^+$ CIK cells in CIK_{IL-2} and CIK_{IL-15} in all three donors

	**$CD3^+CD56^+CIK_{IL-2}$**	**$CD3^+CD56^+CIK_{IL-15}$**
**Donor 1**	99.2%	98.2%
**Donor 2**	99.4%	98.0%
**Donor 3**	96.8%	97.9%
**Average**	98.47%±0.84	98.03%±0.09

Table S2

Differentially expressed miRNAs between CIK_{IL-2} and PBMCs

SystematicName	pvalues	foldchange	IL_2_1_NS	IL_2_2_NS	IL_2_3_NS	IL_2_1	IL_2_2	IL_2_3	C1_NS	C2_NS	C3_NS	C1	C2	C3
hsa-let-7a-5p	0.002321289	0.49141034	11.187136	11.100483	10.821659	P	P	P	12.159533	11.871196	12.159533	P	P	P
hsa-let-7b-5p	7.39E-05	0.0778887	6.7402406	7.079769	7.0410886	P	P	P	10.697974	10.529345	10.697974	P	P	P
hsa-let-7c	3.20E-05	0.11994789	5.5676794	5.9008718	5.6869106	P	P	P	8.89448	8.654416	8.795037	P	P	P
hsa-let-7e-5p	0.000570431	0.00087028	-3.205473	-3.080319	-3.147785	A	A	A	7.2060633	6.4438534	7.279448	P	P	P
hsa-miR-1	7.95E-05	0.00222873	-3.205473	-3.080319	-3.147785	A	A	A	5.9008718	5.4622045	5.5992913	P	P	P
hsa-miR-101-3p	0.026000351	0.00492632	1.5514443	-3.080319	-3.147785	A	A	A	7.996314	7.471844	7.707526	P	P	P
hsa-miR-103a-3p	0.000621321	0.36190369	9.583749	9.277758	9.454361	P	P	P	10.919219	10.985516	10.821659	P	P	P
hsa-miR-1181	0.000136234	172.798189	3.9791927	4.072406	4.27377	P	P	P	-3.3194392	-3.3219283	-3.3162193	A	A	A
hsa-miR-1207-5p	0.000483071	2.56308989	6.924969	7.2188106	7.2060633	P	P	P	5.6103806	5.7690125	5.9008718	P	P	P
hsa-miR-1224-5p	0.027788313	3.56821091	3.9988286	4.1800866	4.468674	P	P	P	1.8071963	1.9811914	3.0562327	P	P	P
hsa-miR-1225-5p	0.017752555	2.70897441	8.331444	8.498981	9.099379	P	P	P	7.079769	7.297641	7.3427973	P	P	P
hsa-miR-1228-3p	0.023859014	2.87875051	3.5811896	4.2020206	4.236219	P	P	P	2.5230556	1.8071963	2.9727118	P	P	P
hsa-miR-1229-5p	0.00121998	3.23564526	6.330645	6.07377	6.053328	P	P	P	4.1800866	4.510607	4.660762	P	P	P
hsa-miR-1234-3p	0.043448917	7.34031515	3.1611705	3.8999321	3.6636858	P	P	P	-0.1576762	-0.77899367	1.8071963	P	P	P
hsa-miR-1234-5p	0.003958922	2.02023698	8.576817	8.727931	8.97648	P	P	P	7.499739	7.846886	7.88882	P	P	P
hsa-miR-1238-3p	0.015175267	13.150972	2.911333	3.4417999	3.3828213	P	P	P	0.3587662	-1.6410956	-0.7646921	P	P	P
hsa-miR-124-3p	0.000275767	4894.0383	9.277758	8.871771	8.576817	P	P	P	-3.3194392	-3.3219283	-3.3162193	A	A	A
hsa-miR-124-5p	0.002856455	125.178794	4.2502084	3.4044702	3.0010812	P	P	P	-3.3194392	-3.3219283	-3.3162193	A	A	A
hsa-miR-1246	1.32E-05	13.5403715	9.87587	9.568089	9.675815	P	P	P	5.876487	5.9008718	6.07377	P	P	P
hsa-miR-1249	0.014119703	3.74318765	3.6754284	4.4452944	4.4806085	P	P	P	1.8204625	1.9624975	2.9575922	P	P	P
hsa-miR-125a-5p	0.001459363	0.00251217	-3.205473	-3.080319	-3.147785	A	A	A	5.5088573	4.7766256	5.9585047	P	P	P
hsa-miR-125b-5p	0.000351855	0.0107473	-3.205473	-3.080319	-3.147785	A	A	A	3.6979222	3.1845312	3.2505643	P	P	P
hsa-miR-126-3p	1.49E-09	0.00012518	-3.205473	-3.080319	-3.147785	A	A	A	9.87587	9.750648	9.830814	P	P	P

hsa-miR-126-5p	1.83E-06	0.00184284	-3.205473	-3.080319	-3.147785	A	A	A	6.029608	5.795355	5.985171	P	P	P
hsa-miR-1260a	0.005174346	3.41998493	8.295967	9.099379	8.6821575	P	P	P	6.7691016	6.803901	7.244012	P	P	P
hsa-miR-1260b	0.015786969	3.87256002	6.878999	7.9665885	7.3909016	P	P	P	5.3887925	5.3887925	5.7690125	P	P	P
hsa-miR-1268a	7.80E-05	4.98250257	7.499739	7.279448	7.279448	P	P	P	4.981756	5.016679	5.1175156	P	P	P
hsa-miR-1268b	0.005016269	2.18126332	5.5211577	5.4821186	5.876487	P	P	P	4.6215453	4.428732	4.4806085	P	P	P
hsa-miR-1273g-3p	0.000315017	2.71256882	12.159533	12.037135	11.871196	P	P	P	10.529345	10.697974	10.529345	P	P	P
hsa-miR-1275	4.26E-05	4.23909198	7.908693	7.803902	8.073952	P	P	P	5.715176	5.9585047	5.864165	P	P	P
hsa-miR-128	9.66E-05	0.39198113	5.334731	5.316743	5.200648	P	P	P	6.6779585	6.6242375	6.606022	P	P	P
hsa-miR-1290	0.000255584	511.867894	7.996314	7.761113	7.4553313	P	P	P	-1.2630036	-1.2086849	-1.2646993	P	P	P
hsa-miR-1305	0.021681533	2.71068975	5.093093	4.3913536	4.2020206	P	P	P	3.1845312	2.947276	3.3672557	P	P	P
hsa-miR-130a-3p	0.000245083	0.00024398	-3.205473	-3.080319	-3.147785	A	A	A	8.795037	9.219326	8.455889	P	P	P
hsa-miR-130b-3p	0.002811744	2.44380343	7.846886	7.4553313	7.707526	P	P	P	6.383481	6.301651	6.4786963	P	P	P
hsa-miR-133b	3.58E-07	0.00660912	-3.205473	-3.080319	-3.147785	A	A	A	4.2020206	4.072406	4.0121217	P	P	P
hsa-miR-134	0.02945955	10.652924	3.8562999	4.27377	4.260568	P	P	P	0.43739635	1.607718	-0.80410147	P	P	P
hsa-miR-135a-3p	0.000788959	57.614246	4.796856	4.9981575	5.316743	P	P	P	-1.213372	-0.20742983	-1.2121661	P	P	P
hsa-miR-140-3p	9.92E-07	0.29625185	6.7691016	6.693791	6.693791	P	P	P	8.498981	8.424062	8.498981	P	P	P
hsa-miR-140-5p	0.002102043	0.22809426	6.012252	5.5088573	5.5676794	P	P	P	7.761113	7.803902	7.9665885	P	P	P
hsa-miR-142-3p	0.0040941	0.32351195	10.529345	10.428829	10.529345	P	P	P	11.871196	12.159533	12.309613	P	P	P
hsa-miR-142-5p	0.008726102	0.28644017	7.576128	7.908693	7.297641	P	P	P	9.401786	9.454361	9.401786	P	P	P
hsa-miR-143-3p	0.000262136	0.00521261	-3.205473	-3.080319	-3.147785	A	A	A	4.7004194	4.1365037	4.428732	P	P	P
hsa-miR-144-3p	0.002278422	0.00069677	-3.205473	-3.080319	-3.147785	A	A	A	7.803902	7.576128	6.187962	P	P	P
hsa-miR-144-5p	0.006867942	0.00508099	-3.205473	-3.080319	-3.147785	A	A	A	4.952126	4.796856	3.010559	P	P	P
hsa-miR-145-5p	8.54E-05	0.00118125	-3.205473	-3.080319	-3.147785	A	A	A	6.831531	6.3576484	6.5153904	P	P	P
hsa-miR-1469	0.003614079	106.369839	2.5230556	3.605049	3.8130388	P	P	P	-3.3194392	-3.3219283	-3.3162193	A	A	A
hsa-miR-146b-5p	0.000992057	5.06967955	9.154404	9.050771	9.050771	P	P	P	6.924969	6.5422354	6.7402406	P	P	P
hsa-miR-148a-3p	0.000582859	0.10216894	4.7004194	4.544902	5.250404	P	P	P	8.248948	7.88882	8.295967	P	P	P

hsa-miR-148b-3p	0.000564592	0.09156793	4.0121217	3.5490105	3.6479452	P	P	P	7.279448	7.2060633	7.1076965	P	P	P
hsa-miR-151a-3p	1.41E-05	0.00130378	-3.205473	-3.080319	-3.147785	A	A	A	6.606022	6.273411	6.41963	P	P	P
hsa-miR-151a-5p	7.46E-05	0.00046955	-3.205473	-3.080319	-3.147785	A	A	A	8.182257	7.707526	7.803902	P	P	P
hsa-miR-151b	9.83E-06	0.0009698	-3.205473	-3.080319	-3.147785	A	A	A	6.978785	6.6779585	6.924969	P	P	P
hsa-miR-155-5p	0.000254818	9.24569663	9.300459	9.87587	9.341949	P	P	P	6.579298	6.2206006	6.1254272	P	P	P
hsa-miR-1587	0.005117168	3.2640919	6.400667	5.932713	5.9008718	P	P	P	4.3709307	4.4862566	4.3073688	P	P	P
hsa-miR-15a-5p	0.000521244	0.22292851	8.373192	7.996314	7.996314	P	P	P	10.255653	10.201173	10.428829	P	P	P
hsa-miR-181a-3p	0.005477757	35.0916258	4.288173	4.218587	4.09926	P	P	P	-1.3771256	-0.23347163	-1.5557616	P	P	P
hsa-miR-181b-5p	0.009453587	4.99486542	6.978785	6.803901	6.924969	P	P	P	4.747054	4.846134	4.0296855	P	P	P
hsa-miR-181c-5p	0.011848202	0.30171826	3.4774175	2.911333	2.7992623	P	P	P	4.8730035	4.747054	4.846134	P	P	P
hsa-miR-181d	0.018163632	9.9740414	3.0318012	3.3038332	3.1611705	P	P	P	0.49663904	-0.20206182	-1.2286452	P	P	P
hsa-miR-185-5p	7.56E-05	0.29954922	6.273411	6.1636677	6.07377	P	P	P	7.9665885	7.996314	7.761113	P	P	P
hsa-miR-186-5p	0.002901167	0.4037683	4.9981575	4.660762	4.582231	P	P	P	6.187962	5.974839	6.029608	P	P	P
hsa-miR-188-5p	0.000168949	4.57456227	5.5088573	5.6713343	5.642425	P	P	P	3.5207887	3.232212	3.4774175	P	P	P
hsa-miR-18b-5p	0.023101609	2.18893331	6.07377	5.3887925	5.6103806	P	P	P	4.717377	4.521168	4.521168	P	P	P
hsa-miR-1914-3p	0.024140425	2.99381991	4.7766256	4.1632457	4.11901	P	P	P	3.010559	2.0270984	3.139044	P	P	P
hsa-miR-1915-3p	0.004982588	2.28732128	6.4917407	6.7691016	6.878999	P	P	P	5.5211577	5.5992913	5.4622045	P	P	P
hsa-miR-192-5p	0.004019097	0.21056202	3.8130388	3.123999	2.8519025	P	P	P	5.795355	5.0731435	5.734473	P	P	P
hsa-miR-193a-3p	0.001484099	0.00590492	-3.205473	-3.080319	-3.147785	A	A	A	3.6205487	4.3413744	4.639106	P	P	P
hsa-miR-193a-5p	0.000570718	0.00541209	-3.205473	-3.080319	-3.147785	A	A	A	4.0296855	4.288173	4.747054	P	P	P
hsa-miR-194-5p	0.04629949	0.03842695	1.0082299	-3.080319	-3.147785	A	A	A	4.428732	3.875716	4.468674	P	P	P
hsa-miR-196b-5p	0.000250336	0.01093878	-3.205473	-3.080319	-3.147785	A	A	A	3.0833638	3.4044702	3.5811896	P	P	P
hsa-miR-197-3p	0.002936592	0.17855365	4.4602914	3.8130388	4.0121217	P	P	P	6.6552534	6.4786963	6.6779585	P	P	P
hsa-miR-197-5p	0.000215113	3.30808641	7.279448	7.182649	7.499739	P	P	P	5.4622045	5.6594257	5.6713343	P	P	P
hsa-miR-1972	2.18E-07	22.8847942	2.947276	3.0973506	3.0318012	P	P	P	-1.4731296	-1.560653	-1.4375921	P	P	P
hsa-miR-1973	0.011145921	5.15658236	7.182649	6.301651	6.147969	P	P	P	4.1632457	4.4806085	4.085778	P	P	P

hsa-miR-199a-3p	4.17E-07	0.0001744	-3.205473	-3.080319	-3.147785	A	A	A	9.454361	9.341949	9.219326	P	P	P
hsa-miR-199a-5p	0.000201654	0.0007857	-3.205473	-3.080319	-3.147785	A	A	A	7.471844	7.135916	6.831531	P	P	P
hsa-miR-199b-5p	1.34E-05	0.0016197	-3.205473	-3.080319	-3.147785	A	A	A	6.07377	5.985171	6.301651	P	P	P
hsa-miR-19a-3p	0.00608335	0.36964265	7.3427973	6.7402406	6.7691016	P	P	P	8.576817	8.455889	8.182257	P	P	P
hsa-miR-20b-5p	0.000399757	2.99069576	9.750648	9.454361	9.521075	P	P	P	8.1229725	8.073952	7.781683	P	P	P
hsa-miR-21-3p	0.015947375	3.88686782	5.3887925	6.187962	6.2206006	P	P	P	3.8999321	4.09926	4.057878	P	P	P
hsa-miR-21-5p	0.001527584	2.02215832	12.653478	12.653478	12.653478	P	P	P	11.7160635	11.59668	11.59668	P	P	P
hsa-miR-210	0.000699196	23.1806594	8.61651	8.1229725	8.295967	P	P	P	4.085778	4.0203185	3.2186894	P	P	P
hsa-miR-215	0.0007821	0.00558478	-3.205473	-3.080319	-3.147785	A	A	A	4.503955	3.8363023	4.573564	P	P	P
hsa-miR-22-3p	0.00241215	0.27238589	7.244012	6.878999	6.6552534	P	P	P	8.97648	8.795037	8.6821575	P	P	P
hsa-miR-22-5p	0.023715726	0.02189685	-0.167622	-3.080319	-3.147785	P	A	A	4.1473293	3.9791927	4.1473293	P	P	P
hsa-miR-221-3p	0.001120434	0.2668069	5.6594257	5.5676794	5.734473	P	P	P	7.419521	7.781683	7.4553313	P	P	P
hsa-miR-221-5p	0.022342942	0.49406415	3.605049	3.1611705	3.010559	P	P	P	4.2502084	4.2502084	4.3913536	P	P	P
hsa-miR-222-3p	0.002363995	2.85896941	6.606022	7.0410886	7.079769	P	P	P	5.2181134	5.4821186	5.5088573	P	P	P
hsa-miR-223-3p	0.000544528	0.00183192	7.135916	6.41963	6.6242375	P	P	P	15.851313	15.851313	15.851313	P	P	P
hsa-miR-223-5p	0.000241626	0.0020776	-3.205473	-3.080319	-3.147785	A	A	A	5.5676794	5.5676794	6.098819	P	P	P
hsa-miR-2392	0.001886489	3.5336389	5.282623	5.200648	5.334731	P	P	P	3.3038332	3.3492763	3.6754284	P	P	P
hsa-miR-23a-3p	0.001866992	0.46156688	10.255653	10.255653	10.255653	P	P	P	11.292758	11.357172	11.458346	P	P	P
hsa-miR-23b-3p	0.000246602	0.07426617	4.846134	4.428732	4.7004194	P	P	P	8.331444	8.498981	8.424062	P	P	P
hsa-miR-26a-5p	0.000165112	0.10874954	8.1229725	8.073952	7.761113	P	P	P	11.100483	11.187136	11.292758	P	P	P
hsa-miR-26b-5p	9.24E-05	0.14832686	8.97648	8.576817	8.654416	P	P	P	11.458346	11.292758	11.7160635	P	P	P
hsa-miR-27a-3p	0.001601928	0.42504812	8.654416	8.795037	8.871771	P	P	P	9.830814	9.976183	10.201173	P	P	P
hsa-miR-27b-3p	0.000169238	0.0986892	4.4806085	4.6215453	4.597137	P	P	P	7.908693	7.908693	7.908693	P	P	P
hsa-miR-28-5p	0.001998209	0.34901796	4.895167	4.521168	4.4862566	P	P	P	6.41963	5.876487	6.147969	P	P	P
hsa-miR-29a-3p	0.00052743	0.48021884	9.675815	9.830814	9.87587	P	P	P	10.821659	10.821659	10.919219	P	P	P
hsa-miR-29b-3p	1.53E-05	0.35402608	7.781683	7.6519704	7.781683	P	P	P	9.277758	9.154404	9.277758	P	P	P

hsa-miR-29c-3p	0.000417107	0.29067303	7.9665885	7.707526	7.846886	P	P	P	9.675815	9.568089	9.634267	P	P	P
hsa-miR-30b-5p	1.35E-05	0.10040736	5.734473	5.4622045	5.5088573	P	P	P	9.050771	8.871771	8.727931	P	P	P
hsa-miR-30c-5p	6.94E-05	0.29182423	6.383481	6.147969	6.1636677	P	P	P	7.88882	8.016529	8.1229725	P	P	P
hsa-miR-30d-5p	4.47E-05	0.13949149	4.544902	4.2502084	4.2502084	P	P	P	7.182649	7.3186646	7.079769	P	P	P
hsa-miR-30e-3p	2.99E-05	0.16107934	4.582231	4.3566866	4.288173	P	P	P	6.955541	6.955541	7.2188106	P	P	P
hsa-miR-30e-5p	0.002547434	0.21893209	6.41963	6.1254272	5.974839	P	P	P	8.424062	8.331444	8.373192	P	P	P
hsa-miR-3125	0.047500013	7.34482352	3.8999321	2.9012508	2.8180554	P	P	P	-1.3099577	-0.6849075	1.577412	P	P	P
hsa-miR-3137	0.000282172	23.9985259	3.201806	2.7992623	3.5662074	P	P	P	-1.6175166	-1.213372	-1.2867527	P	P	P
hsa-miR-3156-5p	0.007144904	4.76342509	3.5662074	2.9575922	3.4774175	P	P	P	1.0082299	0.48188725	1.607718	P	P	P
hsa-miR-3162-5p	0.013356219	2.96418738	7.88882	8.295967	8.775922	P	P	P	6.6242375	7.0410886	6.693791	P	P	P
hsa-miR-3195	0.006153454	3.03496138	5.974839	5.642425	5.4271646	P	P	P	3.7411308	3.935175	4.503955	P	P	P
hsa-miR-3196	0.000226178	2.8692254	5.932713	6.098819	6.187962	P	P	P	4.4806085	4.639106	4.544902	P	P	P
hsa-miR-3198	0.014186478	2.38989772	5.316743	4.8730035	4.7664566	P	P	P	3.6754284	3.7543721	3.8130388	P	P	P
hsa-miR-32-5p	0.000165592	0.01124404	-3.205473	-3.080319	-3.147785	A	A	A	3.5662074	3.123999	3.267211	P	P	P
hsa-miR-320d	0.010925074	0.43599004	6.6242375	6.243199	6.029608	P	P	P	7.6519704	7.419521	7.471844	P	P	P
hsa-miR-320e	0.015527454	0.44280329	6.3576484	5.9585047	5.754958	P	P	P	7.3186646	7.15658	7.182649	P	P	P
hsa-miR-324-5p	0.004634279	0.32676011	4.729522	4.288173	4.796856	P	P	P	6.1254272	6.330645	6.243199	P	P	P
hsa-miR-326	6.64E-05	0.01196555	-3.205473	-3.080319	-3.147785	A	A	A	3.0973506	3.1611705	3.4417999	P	P	P
hsa-miR-331-3p	0.000382123	0.30217748	5.6869106	5.54527	5.5211577	P	P	P	7.297641	7.3427973	7.297641	P	P	P
hsa-miR-335-5p	1.49E-08	0.00222132	-3.205473	-3.080319	-3.147785	A	A	A	5.754958	5.6267304	5.6267304	P	P	P
hsa-miR-338-3p	0.000210067	0.00044139	-3.205473	-3.080319	-3.147785	A	A	A	8.295967	7.61356	8.016529	P	P	P
hsa-miR-340-3p	5.23E-06	0.00229039	-3.205473	-3.080319	-3.147785	A	A	A	5.6267304	5.6103806	5.642425	P	P	P
hsa-miR-340-5p	5.72E-07	0.00063811	-3.205473	-3.080319	-3.147785	A	A	A	7.4553313	7.4553313	7.499739	P	P	P
hsa-miR-342-3p	0.000720645	4.1522712	10.985516	11.187136	10.985516	P	P	P	9.219326	8.97648	8.775922	P	P	P
hsa-miR-342-5p	0.003779614	2.04359536	6.6552534	6.3576484	6.400667	P	P	P	5.6594257	5.2181134	5.4271646	P	P	P
hsa-miR-345-5p	0.000271862	34.9124619	3.7411308	3.5811896	3.875716	P	P	P	-1.3875048	-1.3934426	-1.3830336	P	P	P

hsa-miR-34a-5p	0.002118995	12.0661113	6.301651	7.244012	6.579298	P	P	P	3.232212	2.9259632	3.3260815	P	P	P
hsa-miR-34b-5p	0.001034099	123.875366	3.3828213	4.0428915	3.3672557	P	P	P	-3.3194392	-3.3219283	-3.3162193	A	A	A
hsa-miR-361-5p	0.000588894	0.25584532	5.1175156	4.7766256	4.8730035	P	P	P	6.803901	6.924969	6.955541	P	P	P
hsa-miR-362-3p	1.19E-06	0.0109657	-3.205473	-3.080319	-3.147785	A	A	A	3.4774175	3.267211	3.3492763	P	P	P
hsa-miR-362-5p	0.039799032	0.03586856	0.5563415	-3.080319	-3.147785	A	A	A	4.072406	4.108054	3.7411308	P	P	P
hsa-miR-363-3p	0.001771465	12.5378836	9.601028	9.727392	9.583749	P	P	P	6.273411	6.012252	5.6103806	P	P	P
hsa-miR-3648	0.000737727	114.908994	3.8267286	3.1845312	3.4927819	P	P	P	-3.3194392	-3.3219283	-3.3162193	A	A	A
hsa-miR-3651	0.002566303	2.38983127	7.4553313	7.781683	7.803902	P	P	P	6.330645	6.5153904	6.4438534	P	P	P
hsa-miR-3652	0.000307393	3.3596126	4.3566866	4.536281	4.729522	P	P	P	2.947276	2.835911	2.5966039	P	P	P
hsa-miR-3653	0.00268825	0.43687997	5.0457525	5.3682666	5.3038664	P	P	P	6.5153904	6.41963	6.383481	P	P	P
hsa-miR-365a-3p	0.018185506	0.0065424	0.3587662	-3.080319	-3.147785	A	A	A	5.985171	6.383481	6.400667	P	P	P
hsa-miR-3665	0.000283807	2.91833822	7.3186646	7.576128	7.61356	P	P	P	5.974839	6.07377	5.831571	P	P	P
hsa-miR-3679-5p	0.002397419	2.63645728	6.5422354	6.5422354	6.5153904	P	P	P	5.250404	4.9981575	5.1446013	P	P	P
hsa-miR-374a-5p	0.007286191	0.12597533	6.098819	5.3038664	5.282623	P	P	P	8.654416	8.576817	8.576817	P	P	P
hsa-miR-374b-5p	0.000193451	0.14068493	6.187962	5.7690125	5.8190274	P	P	P	8.6821575	8.6821575	8.923614	P	P	P
hsa-miR-376a-3p	0.007852896	0.00267289	-3.205473	-3.080319	-3.147785	A	A	A	5.8190274	5.864165	3.6205487	P	P	P
hsa-miR-376c-3p	0.007198545	0.00208987	-3.205473	-3.080319	-3.147785	A	A	A	6.147969	6.243199	3.9791927	P	P	P
hsa-miR-3911	0.032317002	10.8750594	3.4044702	3.232212	3.6854112	P	P	P	-1.4567815	-0.7397993	1.0410005	P	P	P
hsa-miR-3940-5p	0.003091185	2.88494273	4.0296855	4.323796	4.428732	P	P	P	2.5438547	2.5438547	3.0712836	P	P	P
hsa-miR-3960	0.039815354	2.82059984	10.697974	10.919219	11.716064	P	P	P	9.568089	9.727392	9.750648	P	P	P
hsa-miR-409-3p	0.022541246	0.0050782	-3.205473	-3.080319	-3.147785	A	A	A	4.846134	5.1175156	1.7524863	P	P	P
hsa-miR-424-5p	0.000159791	0.09444462	4.573564	4.3413744	4.0428915	P	P	P	7.576128	7.499739	8.073952	P	P	P
hsa-miR-425-5p	0.005429064	0.30280939	7.419521	6.978785	7.1076965	P	P	P	8.923614	8.89448	8.89448	P	P	P
hsa-miR-4270	0.012853106	2.37985742	5.54527	6.053328	5.831571	P	P	P	4.260568	4.3709307	4.981756	P	P	P
hsa-miR-4284	0.00052773	4.29217188	10.428829	10.529345	10.919219	P	P	P	8.533429	8.727931	8.331444	P	P	P
hsa-miR-4299	1.06E-05	4.23844691	8.775922	8.61651	8.61651	P	P	P	6.4786963	6.6552534	6.6242375	P	P	P

hsa-miR-4306	0.003093622	0.3136202	6.5153904	6.2206006	6.203362	P	P	P	8.016529	7.9665885	7.996314	P	P	P
hsa-miR-4313	0.015753879	9.09405996	2.7216218	2.9727118	3.3260815	P	P	P	0.5024748	-1.2912214	-0.20742983	P	P	P
hsa-miR-4317	0.002688304	0.28620939	2.7638638	2.8519025	2.911333	P	P	P	4.544902	4.468674	4.895167	P	P	P
hsa-miR-4327	0.025376359	10.7731555	3.6636858	3.0562327	3.6335928	P	P	P	-1.575886	0.43739635	0.49663904	P	P	P
hsa-miR-4417	0.000498343	87.5828511	3.2186894	2.835911	3.3038332	P	P	P	-3.3194392	-3.3219283	-3.3162193	A	A	A
hsa-miR-4428	0.00521767	7.41014914	7.471844	8.182257	8.1229725	P	P	P	5.1446013	5.0457525	5.016679	P	P	P
hsa-miR-4430	7.35E-06	157.399283	3.9523053	3.9637272	4.0203185	P	P	P	-3.3194392	-3.3219283	-3.3162193	A	A	A
hsa-miR-4442	0.005588923	2.27347601	5.754958	5.754958	5.864165	P	P	P	4.521168	4.4452944	4.826829	P	P	P
hsa-miR-4446-3p	0.025420474	23.4367796	4.521168	5.6267304	4.826829	P	P	P	-0.77899367	1.7524863	-1.5280784	P	P	P
hsa-miR-4449	0.010917289	3.03056474	3.010559	3.6335928	3.2505643	P	P	P	1.7724594	1.7328621	1.6575521	P	P	P
hsa-miR-4454	0.009267106	2.4689618	15.475359	15.851313	15.475359	P	P	P	14.307951	14.307951	14.307951	P	P	P
hsa-miR-4455	0.004153925	2.56739427	5.1446013	5.250404	5.6267304	P	P	P	4.09926	3.8562999	4.0203185	P	P	P
hsa-miR-4459	7.60E-05	10.4299116	12.309613	12.309613	12.469452	P	P	P	8.775922	9.050771	9.099379	P	P	P
hsa-miR-4465	0.016780022	8.87405253	5.4622045	5.876487	6.831531	P	P	P	3.0318012	2.9575922	3.0833638	P	P	P
hsa-miR-4478	0.002103999	5.81308045	6.053328	6.400667	6.4917407	P	P	P	3.8363023	3.7719352	3.7543721	P	P	P
hsa-miR-4481	0.000196268	56.878895	4.1632457	4.510607	4.521168	P	P	P	-1.4279094	-1.4709167	-1.3694881	P	P	P
hsa-miR-4485	0.002751077	5.44182368	7.079769	6.4917407	6.4438534	P	P	P	4.108054	4.400464	4.2502084	P	P	P
hsa-miR-4497	0.020108417	4.91473812	5.250404	5.6594257	6.3576484	P	P	P	3.4927819	3.5662074	3.5375905	P	P	P
hsa-miR-4499	0.006163299	2.44169169	4.6796775	4.729522	4.7766256	P	P	P	3.4417999	3.2186894	3.6335928	P	P	P
hsa-miR-4505	0.004635904	3.34091473	8.016529	7.3427973	7.576128	P	P	P	5.831571	6.1254272	5.8190274	P	P	P
hsa-miR-4507	0.00614797	2.87465838	7.0410886	6.579298	6.6779585	P	P	P	5.316743	5.200648	5.250404	P	P	P
hsa-miR-450a-5p	0.000557568	0.00361837	-3.205473	-3.080319	-3.147785	A	A	A	4.660762	4.7664566	5.3682666	P	P	P
hsa-miR-4515	0.007454497	4.30783686	4.3709307	4.597137	4.510607	P	P	P	1.8978319	2.6217492	2.5438547	P	P	P
hsa-miR-451a	0.000759203	2.62E-05	-3.205473	-3.080319	-3.147785	A	A	A	12.469452	12.309613	11.100483	P	P	P
hsa-miR-4530	0.000260235	5.55399459	8.498981	8.923614	8.89448	P	P	P	6.203362	6.4917407	6.2206006	P	P	P
hsa-miR-4532	0.000765635	40.0828726	4.218587	3.9163327	3.6754284	P	P	P	-1.3377944	-1.3985862	-1.3771256	P	P	P

hsa-miR-454-3p	0.016816679	0.42191227	4.952126	4.236219	4.3413744	P	P	P	5.642425	5.734473	5.974839	P	P	P
hsa-miR-4646-5p	0.016821423	2.1125192	3.9163327	4.0296855	4.057878	P	P	P	2.9575922	2.5966039	3.1611705	P	P	P
hsa-miR-4651	0.005475363	81.7913561	3.6335928	3.8363023	4.1365037	P	P	P	-3.3194392	-1.5528152	-3.3162193	A	A	A
hsa-miR-4653-3p	0.006837247	4.49122862	4.817203	4.747054	4.544902	P	P	P	2.0270984	2.6612234	2.8180554	P	P	P
hsa-miR-4656	0.001410466	8.87301665	5.6103806	6.606022	6.273411	P	P	P	3.123999	2.5230556	3.424407	P	P	P
hsa-miR-4665-3p	0.031997395	2.79123972	3.7543721	4.260568	3.9988286	P	P	P	2.8180554	1.7724594	2.7992623	P	P	P
hsa-miR-4667-5p	0.030354337	3.12325958	4.1800866	4.503955	4.503955	P	P	P	2.8519025	2.0079992	3.1845312	P	P	P
hsa-miR-4669	0.001565913	2.60599491	5.7690125	5.9179406	6.098819	P	P	P	4.468674	4.3566866	4.796856	P	P	P
hsa-miR-4672	0.003877764	6.62104625	5.5992913	5.1640263	5.177499	P	P	P	1.9624975	2.7992623	2.8766868	P	P	P
hsa-miR-4689	0.022553825	2.51060025	4.660762	4.796856	5.3887925	P	P	P	3.5375905	3.7274342	3.6979222	P	P	P
hsa-miR-4697-5p	0.000524774	6.48276878	7.2060633	7.61356	7.6519704	P	P	P	5.1640263	4.597137	4.582231	P	P	P
hsa-miR-4698	0.000362943	92.0262269	3.0562327	3.0833638	3.4417999	P	P	P	-3.3194392	-3.3219283	-3.3162193	A	A	A
hsa-miR-4701-3p	0.004509689	22.6422398	3.232212	3.6636858	3.7411308	P	P	P	-1.5009203	-1.5451193	-0.20206182	P	P	P
hsa-miR-4716-3p	0.016973163	5.11388052	4.4168377	3.8267286	3.0562327	P	P	P	1.8319155	0.49663904	1.85082	P	P	P
hsa-miR-4721	0.002691544	5.20510265	7.761113	7.846886	8.248948	P	P	P	5.5992913	5.642425	5.5211577	P	P	P
hsa-miR-4728-5p	0.001090694	4.31013348	6.831531	6.955541	6.714629	P	P	P	4.729522	4.729522	4.729522	P	P	P
hsa-miR-4741	0.000778544	4.42508838	5.3038664	5.864165	5.715176	P	P	P	3.285011	3.4774175	3.7089338	P	P	P
hsa-miR-4743-5p	0.008862913	13.1571818	3.285011	3.8562999	3.424407	P	P	P	-0.20742983	-1.2121661	0.44351065	P	P	P
hsa-miR-4745-5p	0.00049073	55.2182597	4.108054	4.573564	4.3566866	P	P	P	-1.4273754	-1.4509494	-1.4075273	P	P	P
hsa-miR-4746-3p	0.00063467	5.16698256	4.4452944	4.108054	4.3709307	P	P	P	2.0079992	1.9464353	1.8803777	P	P	P
hsa-miR-4758-5p	0.009156088	26.6076833	4.2020206	4.9087005	5.0457525	P	P	P	-1.5229733	0.44351065	0.43739635	P	P	P
hsa-miR-4778-5p	0.00789219	3.25652267	4.6069117	4.826829	4.952126	P	P	P	2.9727118	2.7216218	3.4927819	P	P	P
hsa-miR-4788	0.003276816	2.40815518	4.597137	4.981756	4.9642296	P	P	P	3.4044702	3.4417999	3.875716	P	P	P
hsa-miR-4793-5p	0.010498712	4.64921174	4.186671	4.3073688	4.4168377	P	P	P	1.713976	1.8204625	2.5746284	P	P	P
hsa-miR-486-5p	0.00021166	0.00086144	-3.205473	-3.080319	-3.147785	A	A	A	7.1076965	7.279448	6.6552534	P	P	P
hsa-miR-487b	0.008482072	0.00469636	-3.205473	-3.080319	-3.147785	A	A	A	4.9331126	5.093093	2.9259632	P	P	P

hsa-miR-494	0.000126334	13.3286986	11.716064	11.871196	11.357172	P	P	P	7.846886	8.248948	7.61356	P	P	P
hsa-miR-495-3p	0.015883292	0.00635851	-3.205473	-3.080319	-3.147785	A	A	A	4.573564	4.6796775	1.9811914	P	P	P
hsa-miR-5006-5p	0.00386148	4.4614718	6.714629	7.297641	7.182649	P	P	P	4.817203	4.9642296	4.9981575	P	P	P
hsa-miR-505-5p	0.015475694	0.01853975	-0.739799	-3.080319	-3.147785	A	A	A	3.9791927	3.7962844	3.9163327	P	P	P
hsa-miR-513a-5p	0.021769144	2.80214086	5.9008718	6.6242375	5.9585047	P	P	P	4.796856	4.660762	4.6796775	P	P	P
hsa-miR-513b	0.001171518	87.7825909	4.826829	5.5992913	5.0731435	P	P	P	-1.217311	-1.257201	-1.2841073	P	P	P
hsa-miR-513c-5p	0.002732547	85.237078	4.11901	5.4271646	4.9087005	P	P	P	-1.5651371	-1.5866115	-1.3579133	P	P	P
hsa-miR-5195-3p	0.011991006	4.81178418	4.1473293	4.400464	4.6215453	P	P	P	1.577412	1.85082	2.7216218	P	P	P
hsa-miR-542-3p	0.00084193	0.0076984	-3.205473	-3.080319	-3.147785	A	A	A	3.7543721	3.4927819	4.27377	P	P	P
hsa-miR-542-5p	0.00035274	0.00638393	-3.205473	-3.080319	-3.147785	A	A	A	3.875716	4.0428915	4.4602914	P	P	P
hsa-miR-550a-3-5p	0.000576661	0.01015785	-3.205473	-3.080319	-3.147785	A	A	A	3.3260815	3.201806	3.8267286	P	P	P
hsa-miR-550a-3p	0.000892938	0.01223299	-3.205473	-3.080319	-3.147785	A	A	A	2.911333	3.010559	3.605049	P	P	P
hsa-miR-551b-3p	0.000256801	0.00953882	-3.205473	-3.080319	-3.147785	A	A	A	3.267211	3.6205487	3.7719352	P	P	P
hsa-miR-5581-5p	0.002801576	4.7339799	4.503955	3.9523053	3.7274342	P	P	P	1.9464353	1.577412	2.0079992	P	P	P
hsa-miR-5703	0.00164637	15.5748334	8.248948	7.88882	7.3186646	P	P	P	4.057878	3.6854112	3.9523053	P	P	P
hsa-miR-5739	0.005636512	3.29610755	7.707526	8.248948	8.331444	P	P	P	6.243199	6.400667	6.5422354	P	P	P
hsa-miR-574-3p	9.72E-05	0.00887186	-3.205473	-3.080319	-3.147785	A	A	A	3.6854112	3.4482384	3.8562999	P	P	P
hsa-miR-574-5p	0.013717417	2.57277693	7.61356	7.3186646	7.9665885	P	P	P	6.3576484	6.187962	6.330645	P	P	P
hsa-miR-575	0.002609949	9.21427063	6.6779585	7.419521	7.135916	P	P	P	3.7962844	4.0121217	3.8999321	P	P	P
hsa-miR-5787	0.000629654	8.22340502	8.89448	9.634267	9.401786	P	P	P	6.053328	6.029608	6.714629	P	P	P
hsa-miR-582-5p	0.000498042	0.00107954	-3.205473	-3.080319	-3.147785	A	A	A	7.0410886	6.203362	6.7691016	P	P	P
hsa-miR-584-5p	0.000740021	0.0025861	-3.205473	-3.080319	-3.147785	A	A	A	5.7690125	5.54527	4.9087005	P	P	P
hsa-miR-590-5p	0.015415202	0.18310208	4.236219	3.285011	3.0712836	P	P	P	6.2206006	5.932713	6.053328	P	P	P
hsa-miR-6073	1.16E-06	0.00403769	-3.205473	-3.080319	-3.147785	A	A	A	4.7664566	4.9331126	4.717377	P	P	P
hsa-miR-6085	0.020067871	3.57863314	6.955541	8.016529	7.908693	P	P	P	5.54527	6.053328	5.932713	P	P	P
hsa-miR-6086	0.015225491	3.47699531	4.057878	4.0121217	3.8267286	P	P	P	2.6217492	1.713976	2.0270984	P	P	P

hsa-miR-6087	0.000365142	4.94934749	10.201173	9.976183	10.348797	P	P	P	7.61356	8.1229725	7.846886	P	P	P
hsa-miR-6089	0.004925182	3.04443706	11.100483	10.985516	11.59668	P	P	P	9.521075	9.830814	9.568089	P	P	P
hsa-miR-6124	0.006230302	2.4077376	6.2206006	6.714629	6.7402406	P	P	P	5.282623	5.1446013	5.4821186	P	P	P
hsa-miR-6125	0.000534276	2.20068538	8.727931	8.654416	8.498981	P	P	P	7.3909016	7.6519704	7.419521	P	P	P
hsa-miR-6126	0.011872169	17.7720983	4.468674	3.9791927	4.186671	P	P	P	-1.2086849	0.40997612	0.48188725	P	P	P
hsa-miR-6127	0.021513192	2.42885969	8.871771	8.424062	8.182257	P	P	P	7.2188106	7.182649	7.3186646	P	P	P
hsa-miR-6129	0.004111207	35.5382158	4.27377	5.016679	4.9331126	P	P	P	0.44351065	-0.80410147	-1.3875048	P	P	P
hsa-miR-6131	0.032041827	2.67598524	5.4821186	4.895167	4.6069117	P	P	P	3.6636858	3.6335928	3.5662074	P	P	P
hsa-miR-6132	0.000143815	4.76704412	6.579298	6.6552534	6.803901	P	P	P	4.3566866	4.6215453	4.288173	P	P	P
hsa-miR-622	0.001190788	136.299822	3.3038332	3.7411308	4.1473293	P	P	P	-3.3194392	-3.3219283	-3.3162193	A	A	A
hsa-miR-625-5p	0.001182006	4.95826215	7.803902	7.15658	7.3427973	P	P	P	5.334731	4.9087005	5.177499	P	P	P
hsa-miR-628-5p	0.000592053	0.00535368	-3.205473	-3.080319	-3.147785	A	A	A	4.27377	4.057878	4.7766256	P	P	P
hsa-miR-630	0.000890024	17.2054085	8.795037	8.455889	8.016529	P	P	P	4.510607	4.186671	4.3413744	P	P	P
hsa-miR-642a-3p	0.001933945	5.52496636	9.219326	9.583749	10.038487	P	P	P	6.878999	7.244012	7.3909016	P	P	P
hsa-miR-642b-3p	0.000147829	4.19587341	6.203362	6.330645	6.5422354	P	P	P	4.11901	4.260568	4.4862566	P	P	P
hsa-miR-6510-5p	0.001674803	3.45423553	6.1636677	6.4438534	6.41963	P	P	P	4.536281	4.27377	4.817203	P	P	P
hsa-miR-652-3p	9.53E-05	0.10179196	3.5375905	3.4774175	3.2186894	P	P	P	6.5422354	6.978785	6.579298	P	P	P
hsa-miR-652-5p	7.54E-05	24.3312059	3.1845312	3.5662074	3.7962844	P	P	P	-1.3053184	-0.6937595	-1.2912214	P	P	P
hsa-miR-660-5p	1.08E-05	0.28044043	3.2505643	3.2186894	3.0973506	P	P	P	5.0731435	4.952126	5.0457525	P	P	P
hsa-miR-664a-3p	0.02297722	0.02130996	-0.202062	-3.080319	-3.147785	A	A	A	3.9988286	4.2020206	4.108054	P	P	P
hsa-miR-664b-5p	0.002315558	0.32479551	4.1365037	4.1365037	4.3913536	P	P	P	6.098819	5.831571	5.5676794	P	P	P
hsa-miR-6717-5p	0.0292667	2.50366544	5.3682666	4.817203	4.536281	P	P	P	3.605049	3.5375905	3.7274342	P	P	P
hsa-miR-6722-3p	7.47E-07	26.0187257	3.3492763	3.267211	3.285011	P	P	P	-1.4484043	-1.4363155	-1.3204116	P	P	P
hsa-miR-718	0.002835663	25.1725426	2.6612234	3.4927819	3.267211	P	P	P	-1.4622538	-1.495435	-1.4604862	P	P	P
hsa-miR-762	0.000501581	2.8410351	5.985171	6.012252	6.243199	P	P	P	4.400464	4.544902	4.7664566	P	P	P
hsa-miR-937-5p	0.033916662	2.08919651	4.639106	5.0731435	5.402697	P	P	P	3.9637272	3.9163327	4.1365037	P	P	P

hsa-miR-939-5p	0.010720444	2.03538867	4.8730035	4.9331126	5.1640263	P	P	P	3.6335928	4.1800866	4.0428915	P	P	P
hsa-miR-940	2.10E-05	2.19360505	4.7664566	4.7664566	4.660762	P	P	P	3.5811896	3.5490105	3.6636858	P	P	P
hsa-miR-98-5p	0.010095736	0.3072074	5.715176	5.093093	5.3682666	P	P	P	7.135916	7.079769	7.135916	P	P	P
hsa-miR-99b-5p	0.001914295	0.00565954	-3.205473	-3.080319	-3.147785	A	A	A	4.4602914	3.5811896	4.7004194	P	P	P

Table S3

Differentially expressed miRNAs between CIK_{IL-15} and PBMCs

SystematicName	pvalues	foldchange	IL_15_1_NS	IL_15_2_NS	IL_15_3_NS	IL_15_1	IL_15_2	IL_15_3	C1_NS	C2_NS	C3_NS	C1	C2	C3
hsa-let-7b-5p	0.000502647	0.127358299	7.3909016	7.803902	7.781683	P	P	P	10.697974	10.529345	10.697974	P	P	P
hsa-let-7c	4.06E-05	0.162609097	5.985171	6.187962	6.301651	P	P	P	8.89448	8.654416	8.795037	P	P	P
hsa-let-7e-5p	0.032469373	0.009206075	-3.1578648	-3.1905131	1.7524863	A	A	P	7.2060633	6.4438534	7.279448	P	P	P
hsa-miR-1	0.000200131	0.002179344	-3.1578648	-3.1905131	-3.1796591	A	A	A	5.9008718	5.4622045	5.5992913	P	P	P
hsa-miR-101-3p	0.000167786	0.016535836	1.85082	1.713976	1.8978319	P	P	P	7.996314	7.471844	7.707526	P	P	P
hsa-miR-103a-3p	0.000255888	0.453088678	9.634267	9.830814	9.830814	P	P	P	10.919219	10.985516	10.821659	P	P	P
hsa-miR-1181	0.00164279	227.2641092	3.875716	4.4862566	4.9642296	P	P	P	-3.3194392	-3.3219283	-3.3162193	A	A	A
hsa-miR-1207-5p	0.000660855	2.704993663	7.135916	7.279448	7.182649	P	P	P	5.6103806	5.7690125	5.9008718	P	P	P
hsa-miR-1224-5p	0.029921158	3.738501315	4.186671	4.2020206	4.4806085	P	P	P	1.8071963	1.9811914	3.0562327	P	P	P
hsa-miR-1225-5p	0.004963699	2.161761663	8.533429	8.073952	8.424062	P	P	P	7.079769	7.297641	7.3427973	P	P	P
hsa-miR-1228-3p	0.022670943	3.303069683	4.260568	4.428732	3.9791927	P	P	P	2.5230556	1.8071963	2.9727118	P	P	P
hsa-miR-1229-5p	0.002824988	2.645628215	6.1636677	5.6713343	5.715176	P	P	P	4.1800866	4.510607	4.660762	P	P	P
hsa-miR-1234-3p	0.045614792	8.20434521	3.6636858	3.9163327	3.7089338	P	P	P	-0.1576762	-0.77899367	1.8071963	P	P	P
hsa-miR-1238-3p	0.013241045	13.07564118	3.0833638	3.6335928	2.9575922	P	P	P	0.3587662	-1.6410956	-0.7646921	P	P	P
hsa-miR-124-3p	0.001392451	4692.053367	9.454361	8.871771	7.908693	P	P	P	-3.3194392	-3.3219283	-3.3162193	A	A	A
hsa-miR-1246	0.000943961	10.43806113	9.521075	8.97648	9.454361	P	P	P	5.876487	5.9008718	6.07377	P	P	P
hsa-miR-1249	0.0210137	3.807526577	4.1365037	4.503955	4.1365037	P	P	P	1.8204625	1.9624975	2.9575922	P	P	P
hsa-miR-125a-5p	0.001591981	0.002456504	-3.1578648	-3.1905131	-3.1796591	A	A	A	5.5088573	4.7766256	5.9585047	P	P	P
hsa-miR-125b-5p	0.000581343	0.010509132	-3.1578648	-3.1905131	-3.1796591	A	A	A	3.6979222	3.1845312	3.2505643	P	P	P
hsa-miR-126-3p	2.08E-06	0.000122411	-3.1578648	-3.1905131	-3.1796591	A	A	A	9.87587	9.750648	9.830814	P	P	P
hsa-miR-126-5p	4.70E-05	0.001802003	-3.1578648	-3.1905131	-3.1796591	A	A	A	6.029608	5.795355	5.985171	P	P	P
hsa-miR-1260a	0.001789317	3.563149721	8.654416	8.923614	8.775922	P	P	P	6.7691016	6.803901	7.244012	P	P	P
hsa-miR-1260b	0.003980499	3.382710106	6.955541	7.6519704	7.15658	P	P	P	5.3887925	5.3887925	5.7690125	P	P	P

hsa-miR-1268a	3.48E-06	4.363165863	7.2060633	7.079769	7.2060633	P	P	P	4.981756	5.016679	5.1175156	P	P	P
hsa-miR-1273g-3p	0.000534641	2.210847741	11.871196	11.59668	11.7160635	P	P	P	10.529345	10.697974	10.529345	P	P	P
hsa-miR-1275	0.013507347	3.330797686	7.996314	7.182649	7.4553313	P	P	P	5.715176	5.9585047	5.864165	P	P	P
hsa-miR-128	0.012024996	0.42225476	5.3887925	5.6267304	5.1175156	P	P	P	6.6779585	6.6242375	6.606022	P	P	P
hsa-miR-1290	0.000641777	410.6846665	7.846886	7.1076965	7.244012	P	P	P	-1.2630036	-1.2086849	-1.2646993	P	P	P
hsa-miR-1304-3p	0.018318463	29.33932546	3.4044702	3.5662074	3.0712836	P	P	P	-1.5805372	-3.3219283	-0.69722164	A	A	A
hsa-miR-1305	0.001479499	2.815545797	4.6215453	4.582231	4.796856	P	P	P	3.1845312	2.947276	3.3672557	P	P	P
hsa-miR-130a-3p	0.000330732	0.00023857	-3.1578648	-3.1905131	-3.1796591	A	A	A	8.795037	9.219326	8.455889	P	P	P
hsa-miR-130b-3p	8.31E-05	2.399512552	7.61356	7.761113	7.576128	P	P	P	6.383481	6.301651	6.4786963	P	P	P
hsa-miR-132-3p	0.037364789	2.514890906	4.729522	5.5088573	5.282623	P	P	P	3.935175	3.139044	4.323796	P	P	P
hsa-miR-133b	3.74E-05	0.006462663	-3.1578648	-3.1905131	-3.1796591	A	A	A	4.2020206	4.072406	4.0121217	P	P	P
hsa-miR-134	0.033130516	10.24800534	4.0203185	4.218587	4.0121217	P	P	P	0.43739635	1.607718	-0.80410147	P	P	P
hsa-miR-135a-3p	0.000389537	52.07285121	4.846134	4.536281	5.250404	P	P	P	-1.213372	-0.20742983	-1.2121661	P	P	P
hsa-miR-140-3p	1.47E-06	0.311641114	6.803901	6.831531	6.7402406	P	P	P	8.498981	8.424062	8.498981	P	P	P
hsa-miR-140-5p	4.76E-05	0.242249825	5.754958	5.876487	5.7690125	P	P	P	7.761113	7.803902	7.9665885	P	P	P
hsa-miR-142-3p	0.001518483	0.356413737	10.348797	10.821659	10.697974	P	P	P	11.871196	12.159533	12.309613	P	P	P
hsa-miR-142-5p	0.011914822	0.306810511	7.3427973	8.016529	7.707526	P	P	P	9.401786	9.454361	9.401786	P	P	P
hsa-miR-143-3p	0.000440575	0.005097096	-3.1578648	-3.1905131	-3.1796591	A	A	A	4.7004194	4.1365037	4.428732	P	P	P
hsa-miR-144-3p	0.002357825	0.000681334	-3.1578648	-3.1905131	-3.1796591	A	A	A	7.803902	7.576128	6.187962	P	P	P
hsa-miR-144-5p	0.006946764	0.00496839	-3.1578648	-3.1905131	-3.1796591	A	A	A	4.952126	4.796856	3.010559	P	P	P
hsa-miR-145-5p	0.00019181	0.001155078	-3.1578648	-3.1905131	-3.1796591	A	A	A	6.831531	6.3576484	6.5153904	P	P	P
hsa-miR-146b-5p	0.000550488	4.552750833	9.219326	8.795037	8.727931	P	P	P	6.924969	6.5422354	6.7402406	P	P	P
hsa-miR-148a-3p	5.11E-05	0.104761835	4.660762	5.016679	4.9981575	P	P	P	8.248948	7.88882	8.295967	P	P	P
hsa-miR-148b-3p	1.91E-05	0.10378949	3.9791927	4.0296855	3.7719352	P	P	P	7.279448	7.2060633	7.1076965	P	P	P
hsa-miR-150-5p	0.005103751	2.103160329	12.159533	12.037135	12.309613	P	P	P	11.357172	10.919219	10.985516	P	P	P
hsa-miR-151a-3p	8.66E-05	0.001274889	-3.1578648	-3.1905131	-3.1796591	A	A	A	6.606022	6.273411	6.41963	P	P	P

hsa-miR-151a-5p	0.000161115	0.000459146	-3.1578648	-3.1905131	-3.1796591	A	A	A	8.182257	7.707526	7.803902	P	P	P
hsa-miR-151b	7.24E-05	0.000948314	-3.1578648	-3.1905131	-3.1796591	A	A	A	6.978785	6.6779585	6.924969	P	P	P
hsa-miR-155-5p	0.001286929	7.929313917	8.923614	9.727392	9.154404	P	P	P	6.579298	6.2206006	6.1254272	P	P	P
hsa-miR-1587	0.005336488	2.301711664	5.831571	5.402697	5.5088573	P	P	P	4.3709307	4.4862566	4.3073688	P	P	P
hsa-miR-15a-5p	9.13E-05	0.284894905	8.331444	8.61651	8.498981	P	P	P	10.255653	10.201173	10.428829	P	P	P
hsa-miR-181a-3p	0.004429328	35.62672836	4.0428915	4.3566866	4.260568	P	P	P	-1.3771256	-0.23347163	-1.5557616	P	P	P
hsa-miR-181b-5p	0.002339249	5.500473256	7.244012	6.693791	7.135916	P	P	P	4.747054	4.846134	4.0296855	P	P	P
hsa-miR-181c-5p	0.000825538	0.238202243	2.8766868	2.5438547	2.8180554	P	P	P	4.8730035	4.747054	4.846134	P	P	P
hsa-miR-185-5p	7.59E-05	0.302469081	6.1254272	6.301651	6.1254272	P	P	P	7.9665885	7.996314	7.761113	P	P	P
hsa-miR-186-5p	0.013094262	0.441635272	5.2181134	4.660762	4.717377	P	P	P	6.187962	5.974839	6.029608	P	P	P
hsa-miR-188-5p	0.000202848	3.581518439	5.282623	5.1446013	5.334731	P	P	P	3.5207887	3.232212	3.4774175	P	P	P
hsa-miR-18b-5p	0.000952016	2.053699111	5.6713343	5.642425	5.5676794	P	P	P	4.717377	4.521168	4.521168	P	P	P
hsa-miR-1914-3p	0.037170565	2.86238382	4.27377	4.1632457	4.503955	P	P	P	3.010559	2.0270984	3.139044	P	P	P
hsa-miR-1915-3p	0.00389061	2.238300029	6.5153904	6.6552534	6.878999	P	P	P	5.5211577	5.5992913	5.4622045	P	P	P
hsa-miR-192-5p	0.021604222	0.261906201	4.057878	3.8130388	2.7216218	P	P	P	5.795355	5.0731435	5.734473	P	P	P
hsa-miR-193a-3p	0.001660674	0.005774062	-3.1578648	-3.1905131	-3.1796591	A	A	A	3.6205487	4.3413744	4.639106	P	P	P
hsa-miR-193a-5p	0.000757719	0.005292153	-3.1578648	-3.1905131	-3.1796591	A	A	A	4.0296855	4.288173	4.747054	P	P	P
hsa-miR-196b-5p	0.000472808	0.010696376	-3.1578648	-3.1905131	-3.1796591	A	A	A	3.0833638	3.4044702	3.5811896	P	P	P
hsa-miR-197-3p	4.71E-05	0.214778149	4.3913536	4.521168	4.236219	P	P	P	6.6552534	6.4786963	6.6779585	P	P	P
hsa-miR-197-5p	0.009987258	4.385486799	7.2188106	7.781683	8.073952	P	P	P	5.4622045	5.6594257	5.6713343	P	P	P
hsa-miR-1972	0.000409203	30.31731237	3.139044	3.5811896	3.5375905	P	P	P	-1.4731296	-1.560653	-1.4375921	P	P	P
hsa-miR-1973	0.011670585	9.517806063	6.693791	7.3427973	8.1229725	P	P	P	4.1632457	4.4806085	4.085778	P	P	P
hsa-miR-199a-3p	2.09E-05	0.000170531	-3.1578648	-3.1905131	-3.1796591	A	A	A	9.454361	9.341949	9.219326	P	P	P
hsa-miR-199a-5p	0.000310163	0.000768286	-3.1578648	-3.1905131	-3.1796591	A	A	A	7.471844	7.135916	6.831531	P	P	P
hsa-miR-199b-5p	8.83E-05	0.001583806	-3.1578648	-3.1905131	-3.1796591	A	A	A	6.07377	5.985171	6.301651	P	P	P
hsa-miR-19a-3p	0.003350557	0.327026588	6.831531	6.7402406	6.831531	P	P	P	8.576817	8.455889	8.182257	P	P	P

hsa-miR-20b-5p	0.001370883	3.008911008	9.675815	9.521075	9.568089	P	P	P	8.1229725	8.073952	7.781683	P	P	P
hsa-miR-21-3p	0.034301393	2.194802516	4.6796775	5.4622045	5.2181134	P	P	P	3.8999321	4.09926	4.057878	P	P	P
hsa-miR-210	0.000452156	27.25060661	8.373192	8.89448	8.455889	P	P	P	4.085778	4.0203185	3.2186894	P	P	P
hsa-miR-215	0.000968845	0.005461015	-3.1578648	-3.1905131	-3.1796591	A	A	A	4.503955	3.8363023	4.573564	P	P	P
hsa-miR-22-3p	0.000459163	0.288806068	7.0410886	7.2188106	6.803901	P	P	P	8.97648	8.795037	8.6821575	P	P	P
hsa-miR-22-5p	3.75E-05	0.006481842	-3.1578648	-3.1905131	-3.1796591	A	A	A	4.1473293	3.9791927	4.1473293	P	P	P
hsa-miR-221-3p	0.007641454	0.235548897	4.9331126	5.864165	5.4821186	P	P	P	7.419521	7.781683	7.4553313	P	P	P
hsa-miR-222-3p	0.004817641	2.298933986	6.301651	6.803901	6.6779585	P	P	P	5.2181134	5.4821186	5.5088573	P	P	P
hsa-miR-223-3p	0.000382942	0.001571272	6.878999	6.330645	6.330645	P	P	P	15.851313	15.851313	15.851313	P	P	P
hsa-miR-223-5p	0.000379948	0.002031557	-3.1578648	-3.1905131	-3.1796591	A	A	A	5.5676794	5.5676794	6.098819	P	P	P
hsa-miR-2392	0.018214191	2.731528497	5.3682666	4.597137	4.597137	P	P	P	3.3038332	3.3492763	3.6754284	P	P	P
hsa-miR-23b-3p	3.77E-05	0.100756318	5.200648	5.177499	4.9331126	P	P	P	8.331444	8.498981	8.424062	P	P	P
hsa-miR-26a-5p	0.001234675	0.155439923	8.775922	8.455889	8.248948	P	P	P	11.100483	11.187136	11.292758	P	P	P
hsa-miR-26b-5p	0.000322099	0.181067633	9.099379	9.099379	8.89448	P	P	P	11.458346	11.292758	11.7160635	P	P	P
hsa-miR-27b-3p	0.002282611	0.112388538	4.4602914	4.981756	4.7766256	P	P	P	7.908693	7.908693	7.908693	P	P	P
hsa-miR-28-5p	0.004349819	0.380287575	4.8730035	4.817203	4.6069117	P	P	P	6.41963	5.876487	6.147969	P	P	P
hsa-miR-29a-3p	0.00215004	0.488894116	9.750648	9.976183	9.727392	P	P	P	10.821659	10.821659	10.919219	P	P	P
hsa-miR-29b-1-5p	0.000419957	0.406477188	3.1611705	2.9727118	3.0318012	P	P	P	4.236219	4.3073688	4.510607	P	P	P
hsa-miR-29b-3p	0.007234628	0.36236589	7.471844	7.996314	7.803902	P	P	P	9.277758	9.154404	9.277758	P	P	P
hsa-miR-29c-3p	0.001735926	0.325014866	7.9665885	8.182257	7.846886	P	P	P	9.675815	9.568089	9.634267	P	P	P
hsa-miR-29c-5p	0.005112767	0.165942959	2.6217492	1.7724594	1.7328621	P	P	P	4.582231	4.536281	4.9331126	P	P	P
hsa-miR-301a-3p	0.000983029	0.160972886	2.9012508	2.5966039	2.7992623	P	P	P	5.4271646	5.402697	5.3887925	P	P	P
hsa-miR-30b-5p	0.000466227	0.176431727	6.330645	6.4438534	6.383481	P	P	P	9.050771	8.871771	8.727931	P	P	P
hsa-miR-30c-5p	0.001197719	0.369457913	6.606022	6.579298	6.5422354	P	P	P	7.88882	8.016529	8.1229725	P	P	P
hsa-miR-30d-5p	3.61E-05	0.251580629	5.316743	5.2181134	5.0731435	P	P	P	7.182649	7.3186646	7.079769	P	P	P
hsa-miR-30e-3p	6.00E-05	0.173711349	4.544902	4.6796775	4.323796	P	P	P	6.955541	6.955541	7.2188106	P	P	P

hsa-miR-30e-5p	8.45E-06	0.274937706	6.4438534	6.5153904	6.579298	P	P	P	8.424062	8.331444	8.373192	P	P	P
hsa-miR-3125	0.047072781	9.223981839	3.8363023	3.3260815	3.6636858	P	P	P	-1.3099577	-0.6849075	1.577412	P	P	P
hsa-miR-3135b	0.00429488	2.150239493	6.012252	5.715176	6.1636677	P	P	P	4.895167	4.6069117	5.0731435	P	P	P
hsa-miR-3156-5p	0.007875109	5.522978402	3.6479452	3.285011	3.7411308	P	P	P	1.0082299	0.48188725	1.607718	P	P	P
hsa-miR-3162-5p	0.001636948	2.554564473	8.182257	7.908693	8.331444	P	P	P	6.6242375	7.0410886	6.693791	P	P	P
hsa-miR-3195	0.012453363	2.898999129	5.5676794	5.5211577	5.795355	P	P	P	3.7411308	3.935175	4.503955	P	P	P
hsa-miR-3196	2.07E-05	2.851052576	5.974839	6.1254272	6.098819	P	P	P	4.4806085	4.639106	4.544902	P	P	P
hsa-miR-3198	0.013607711	2.267091162	4.952126	4.6215453	5.1640263	P	P	P	3.6754284	3.7543721	3.8130388	P	P	P
hsa-miR-32-5p	0.000376641	0.010994871	-3.1578648	-3.1905131	-3.1796591	A	A	A	3.5662074	3.123999	3.267211	P	P	P
hsa-miR-320d	0.012334266	0.485827049	6.7691016	6.383481	6.2206006	P	P	P	7.6519704	7.419521	7.471844	P	P	P
hsa-miR-320e	0.017755392	0.488024954	6.4917407	6.07377	5.932713	P	P	P	7.3186646	7.15658	7.182649	P	P	P
hsa-miR-324-5p	0.003917608	0.364525034	4.4806085	4.9087005	4.9087005	P	P	P	6.1254272	6.330645	6.243199	P	P	P
hsa-miR-326	0.000245166	0.011700386	-3.1578648	-3.1905131	-3.1796591	A	A	A	3.0973506	3.1611705	3.4417999	P	P	P
hsa-miR-331-3p	1.17E-05	0.308426891	5.6103806	5.6103806	5.6267304	P	P	P	7.297641	7.3427973	7.297641	P	P	P
hsa-miR-335-5p	9.67E-06	0.002172091	-3.1578648	-3.1905131	-3.1796591	A	A	A	5.754958	5.6267304	5.6267304	P	P	P
hsa-miR-338-3p	0.000306202	0.000431609	-3.1578648	-3.1905131	-3.1796591	A	A	A	8.295967	7.61356	8.016529	P	P	P
hsa-miR-340-3p	3.25E-11	0.002239636	-3.1578648	-3.1905131	-3.1796591	A	A	A	5.6267304	5.6103806	5.642425	P	P	P
hsa-miR-340-5p	9.66E-10	0.00062397	-3.1578648	-3.1905131	-3.1796591	A	A	A	7.4553313	7.4553313	7.499739	P	P	P
hsa-miR-342-3p	0.000525204	3.922375312	10.821659	11.100483	10.985516	P	P	P	9.219326	8.97648	8.775922	P	P	P
hsa-miR-345-5p	0.00110546	22.95823187	3.2505643	2.8180554	3.285011	P	P	P	-1.3875048	-1.3934426	-1.3830336	P	P	P
hsa-miR-34a-5p	0.006493853	12.13402519	6.147969	7.3909016	6.4786963	P	P	P	3.232212	2.9259632	3.3260815	P	P	P
hsa-miR-361-5p	0.002745561	0.368351992	5.250404	5.6594257	5.4271646	P	P	P	6.803901	6.924969	6.955541	P	P	P
hsa-miR-362-3p	6.05E-05	0.010722693	-3.1578648	-3.1905131	-3.1796591	A	A	A	3.4774175	3.267211	3.3492763	P	P	P
hsa-miR-362-5p	0.000244726	0.00699718	-3.1578648	-3.1905131	-3.1796591	A	A	A	4.072406	4.108054	3.7411308	P	P	P
hsa-miR-363-3p	0.000844543	11.44751378	9.583749	9.583749	9.341949	P	P	P	6.273411	6.012252	5.6103806	P	P	P
hsa-miR-3651	0.000460208	2.343234637	7.761113	7.707526	7.499739	P	P	P	6.330645	6.5153904	6.4438534	P	P	P

hsa-miR-3652	0.000517818	3.074635023	4.510607	4.1800866	4.544902	P	P	P	2.947276	2.835911	2.5966039	P	P	P
hsa-miR-3663-3p	0.006360913	0.461211506	5.4271646	5.5676794	5.6594257	P	P	P	6.400667	6.693791	6.878999	P	P	P
hsa-miR-3665	0.000252528	4.031333623	8.016529	8.1229725	7.761113	P	P	P	5.974839	6.07377	5.831571	P	P	P
hsa-miR-3679-5p	0.006839172	2.039021431	6.41963	5.974839	6.053328	P	P	P	5.250404	4.9981575	5.1446013	P	P	P
hsa-miR-371a-5p	0.000302523	107.4106522	3.201806	3.4482384	3.605049	P	P	P	-3.3194392	-3.3219283	-3.3162193	A	A	A
hsa-miR-374a-5p	5.64E-05	0.140784728	5.876487	5.7690125	5.6713343	P	P	P	8.654416	8.576817	8.576817	P	P	P
hsa-miR-374b-5p	2.69E-05	0.167762778	6.273411	6.2206006	6.07377	P	P	P	8.6821575	8.6821575	8.923614	P	P	P
hsa-miR-376a-3p	0.007899754	0.002613659	-3.1578648	-3.1905131	-3.1796591	A	A	A	5.8190274	5.864165	3.6205487	P	P	P
hsa-miR-376c-3p	0.00724674	0.002043556	-3.1578648	-3.1905131	-3.1796591	A	A	A	6.147969	6.243199	3.9791927	P	P	P
hsa-miR-3940-5p	0.001299302	3.731245896	4.3566866	4.846134	4.6796775	P	P	P	2.5438547	2.5438547	3.0712836	P	P	P
hsa-miR-3960	8.45E-05	2.563758864	11.100483	10.919219	11.100483	P	P	P	9.568089	9.727392	9.750648	P	P	P
hsa-miR-409-3p	0.022443028	0.004965669	-3.1578648	-3.1905131	-3.1796591	A	A	A	4.846134	5.1175156	1.7524863	P	P	P
hsa-miR-424-5p	0.002941437	0.054026943	3.1845312	4.186671	2.8766868	P	P	P	7.576128	7.499739	8.073952	P	P	P
hsa-miR-425-5p	0.001322222	0.378859402	7.419521	7.61356	7.471844	P	P	P	8.923614	8.89448	8.89448	P	P	P
hsa-miR-4284	0.000365581	3.905981682	10.697974	10.255653	10.529345	P	P	P	8.533429	8.727931	8.331444	P	P	P
hsa-miR-4299	0.005697348	3.867111382	8.248948	8.424062	8.871771	P	P	P	6.4786963	6.6552534	6.6242375	P	P	P
hsa-miR-4306	0.005333121	0.287401041	6.383481	6.243199	5.9179406	P	P	P	8.016529	7.9665885	7.996314	P	P	P
hsa-miR-4313	0.009810144	14.15637293	3.3260815	3.9791927	3.6205487	P	P	P	0.5024748	-1.2912214	-0.20742983	P	P	P
hsa-miR-4317	0.000536626	0.282405532	2.9575922	2.911333	2.5746284	P	P	P	4.544902	4.468674	4.895167	P	P	P
hsa-miR-4327	0.023499039	13.92877633	3.7089338	3.6854112	4.108054	P	P	P	-1.575886	0.43739635	0.49663904	P	P	P
hsa-miR-4428	0.001579261	7.983307181	7.803902	8.331444	8.016529	P	P	P	5.1446013	5.0457525	5.016679	P	P	P
hsa-miR-4430	0.001148079	223.0690673	3.9637272	4.468674	4.8730035	P	P	P	-3.3194392	-3.3219283	-3.3162193	A	A	A
hsa-miR-4433-5p	0.006804983	4.785175079	3.285011	4.3913536	3.6854112	P	P	P	1.6791455	1.0082299	1.9624975	P	P	P
hsa-miR-4446-3p	0.020974547	20.46243629	4.9981575	5.54527	3.2186894	P	P	P	-0.77899367	1.7524863	-1.5280784	P	P	P
hsa-miR-4449	0.012711868	3.050114876	3.5662074	3.424407	2.9259632	P	P	P	1.7724594	1.7328621	1.6575521	P	P	P
hsa-miR-4454	0.009267106	2.468961802	15.475359	15.851313	15.475359	P	P	P	14.307951	14.307951	14.307951	P	P	P

hsa-miR-4455	0.007227337	2.29904625	5.4622045	4.9331126	5.1446013	P	P	P	4.09926	3.8562999	4.0203185	P	P	P
hsa-miR-4459	0.001494661	9.731053644	12.653478	11.871196	12.159533	P	P	P	8.775922	9.050771	9.099379	P	P	P
hsa-miR-4465	0.016383131	12.2447123	5.715176	6.4786963	7.297641	P	P	P	3.0318012	2.9575922	3.0833638	P	P	P
hsa-miR-4478	0.000288691	4.980897403	6.029608	6.029608	6.243199	P	P	P	3.8363023	3.7719352	3.7543721	P	P	P
hsa-miR-4481	0.000370249	44.35642738	4.3073688	3.8999321	3.8999321	P	P	P	-1.4279094	-1.4709167	-1.3694881	P	P	P
hsa-miR-4485	0.012474786	8.986726064	6.6552534	7.3186646	7.996314	P	P	P	4.108054	4.400464	4.2502084	P	P	P
hsa-miR-4497	0.002929645	5.126465661	5.6869106	5.795355	6.147969	P	P	P	3.4927819	3.5662074	3.5375905	P	P	P
hsa-miR-4499	0.00163931	2.356059854	4.597137	4.573564	4.846134	P	P	P	3.4417999	3.2186894	3.6335928	P	P	P
hsa-miR-4505	0.000614925	2.733450139	7.576128	7.2060633	7.3427973	P	P	P	5.831571	6.1254272	5.8190274	P	P	P
hsa-miR-4507	0.002791593	2.223113577	6.579298	6.273411	6.3576484	P	P	P	5.316743	5.200648	5.250404	P	P	P
hsa-miR-450a-5p	0.000722762	0.003538187	-3.1578648	-3.1905131	-3.1796591	A	A	A	4.660762	4.7664566	5.3682666	P	P	P
hsa-miR-4515	0.00163244	5.724960328	4.503955	4.7766256	5.316743	P	P	P	1.8978319	2.6217492	2.5438547	P	P	P
hsa-miR-451a	0.000809947	2.56E-05	-3.1578648	-3.1905131	-3.1796591	A	A	A	12.469452	12.309613	11.100483	P	P	P
hsa-miR-4530	0.000154275	4.645828564	8.295967	8.576817	8.6821575	P	P	P	6.203362	6.4917407	6.2206006	P	P	P
hsa-miR-4532	0.000989012	41.74027294	3.8562999	3.7719352	4.3413744	P	P	P	-1.3377944	-1.3985862	-1.3771256	P	P	P
hsa-miR-454-3p	0.038721641	0.440721062	5.1175156	4.3413744	4.186671	P	P	P	5.642425	5.734473	5.974839	P	P	P
hsa-miR-4649-3p	0.024325576	11.75743531	3.3492763	4.0203185	3.0562327	P	P	P	-0.7249392	-1.5651371	1.0082299	P	P	P
hsa-miR-4651	0.002448855	98.52283175	3.7274342	3.9523053	4.6215453	P	P	P	-3.3194392	-1.5528152	-3.3162193	A	A	A
hsa-miR-4653-3p	0.002038724	5.832406845	4.639106	4.952126	5.5211577	P	P	P	2.0270984	2.6612234	2.8180554	P	P	P
hsa-miR-4656	0.005885517	7.504842541	5.9008718	6.5422354	5.177499	P	P	P	3.123999	2.5230556	3.424407	P	P	P
hsa-miR-4665-3p	0.02772565	3.359137564	4.218587	4.4602914	4.1632457	P	P	P	2.8180554	1.7724594	2.7992623	P	P	P
hsa-miR-4667-5p	0.0387842	2.992201379	4.4452944	4.323796	4.2502084	P	P	P	2.8519025	2.0079992	3.1845312	P	P	P
hsa-miR-4669	0.012619507	2.228213036	5.864165	5.282623	5.9008718	P	P	P	4.468674	4.3566866	4.796856	P	P	P
hsa-miR-4672	0.002609065	11.06956077	5.3038664	5.985171	6.6242375	P	P	P	1.9624975	2.7992623	2.8766868	P	P	P
hsa-miR-4697-5p	0.002759522	5.121821908	7.279448	7.471844	6.606022	P	P	P	5.1640263	4.597137	4.582231	P	P	P
hsa-miR-4698	3.92E-05	94.28210062	3.3038332	3.2505643	3.1611705	P	P	P	-3.3194392	-3.3219283	-3.3162193	A	A	A

hsa-miR-4701-3p	0.005552946	21.90344739	3.7543721	3.3038332	3.4482384	P	P	P	-1.5009203	-1.5451193	-0.20206182	P	P	P
hsa-miR-4713-3p	0.028209411	2.166211119	5.5088573	5.3038664	6.012252	P	P	P	4.288173	4.981756	4.1632457	P	P	P
hsa-miR-4716-3p	0.020551599	6.424023577	4.085778	4.09926	4.3913536	P	P	P	1.8319155	0.49663904	1.85082	P	P	P
hsa-miR-4721	0.010433738	4.078274592	7.707526	7.15658	7.88882	P	P	P	5.5992913	5.642425	5.5211577	P	P	P
hsa-miR-4728-5p	0.00096172	4.820702283	6.924969	7.135916	6.924969	P	P	P	4.729522	4.729522	4.729522	P	P	P
hsa-miR-4739	0.035248866	2.890688004	7.15658	7.846886	6.7691016	P	P	P	5.9585047	5.754958	5.6594257	P	P	P
hsa-miR-4741	0.003462788	3.595493201	5.334731	5.316743	5.3887925	P	P	P	3.285011	3.4774175	3.7089338	P	P	P
hsa-miR-4743-5p	0.018463195	10.8144832	3.6854112	3.6636858	1.6791455	P	P	P	-0.20742983	-1.2121661	0.44351065	P	P	P
hsa-miR-4745-5p	1.55E-05	55.27869095	4.323796	4.4452944	4.3073688	P	P	P	-1.4273754	-1.4509494	-1.4075273	P	P	P
hsa-miR-4746-3p	0.024781197	8.56917961	4.108054	4.796856	5.754958	P	P	P	2.0079992	1.9464353	1.8803777	P	P	P
hsa-miR-4758-5p	0.014791708	21.23220257	4.717377	4.1365037	4.400464	P	P	P	-1.5229733	0.44351065	0.43739635	P	P	P
hsa-miR-4778-5p	0.014313881	2.341090755	4.536281	4.0428915	4.3566866	P	P	P	2.9727118	2.7216218	3.4927819	P	P	P
hsa-miR-4788	0.003619134	2.523486313	4.747054	4.9981575	5.016679	P	P	P	3.4044702	3.4417999	3.875716	P	P	P
hsa-miR-4793-5p	0.007302607	4.997181945	4.3709307	4.260568	4.582231	P	P	P	1.713976	1.8204625	2.5746284	P	P	P
hsa-miR-486-5p	0.000322779	0.000842346	-3.1578648	-3.1905131	-3.1796591	A	A	A	7.1076965	7.279448	6.6552534	P	P	P
hsa-miR-487b	0.008537409	0.00459229	-3.1578648	-3.1905131	-3.1796591	A	A	A	4.9331126	5.093093	2.9259632	P	P	P
hsa-miR-494	0.001517346	21.88567354	11.59668	12.653478	12.653478	P	P	P	7.846886	8.248948	7.61356	P	P	P
hsa-miR-495-3p	0.01585915	0.006217608	-3.1578648	-3.1905131	-3.1796591	A	A	A	4.573564	4.6796775	1.9811914	P	P	P
hsa-miR-5006-5p	0.001487157	4.869267559	7.182649	7.4553313	6.955541	P	P	P	4.817203	4.9642296	4.9981575	P	P	P
hsa-miR-505-3p	0.000951095	0.13725918	2.9727118	2.7992623	2.6612234	P	P	P	5.6869106	5.6713343	5.6869106	P	P	P
hsa-miR-505-5p	3.47E-05	0.007415705	-3.1578648	-3.1905131	-3.1796591	A	A	A	3.9791927	3.7962844	3.9163327	P	P	P
hsa-miR-513a-5p	0.011262802	2.922965337	6.07377	6.606022	6.029608	P	P	P	4.796856	4.660762	4.6796775	P	P	P
hsa-miR-513b	0.001543973	93.26302563	5.0731435	5.754958	4.895167	P	P	P	-1.217311	-1.257201	-1.2841073	P	P	P
hsa-miR-513c-5p	0.002317031	79.53736089	4.6069117	5.3887925	4.1800866	P	P	P	-1.5651371	-1.5866115	-1.3579133	P	P	P
hsa-miR-5194	0.002060193	21.82183333	3.7411308	3.0562327	3.7543721	P	P	P	-1.5887748	-1.2646993	-0.21133436	P	P	P
hsa-miR-5195-3p	0.017259227	3.670589698	4.236219	3.7089338	4.0428915	P	P	P	1.577412	1.85082	2.7216218	P	P	P

hsa-miR-532-5p	4.05E-05	0.187611291	2.0270984	1.85082	2.0079992	P	P	P	4.4862566	4.4168377	4.218587	P	P	P
hsa-miR-542-3p	0.001049635	0.007527799	-3.1578648	-3.1905131	-3.1796591	A	A	A	3.7543721	3.4927819	4.27377	P	P	P
hsa-miR-542-5p	0.000546953	0.006242459	-3.1578648	-3.1905131	-3.1796591	A	A	A	3.875716	4.0428915	4.4602914	P	P	P
hsa-miR-550a-3-5p	0.000807605	0.009932749	-3.1578648	-3.1905131	-3.1796591	A	A	A	3.3260815	3.201806	3.8267286	P	P	P
hsa-miR-550a-3p	0.001138553	0.011961905	-3.1578648	-3.1905131	-3.1796591	A	A	A	2.911333	3.010559	3.605049	P	P	P
hsa-miR-551b-3p	0.000470427	0.009327434	-3.1578648	-3.1905131	-3.1796591	A	A	A	3.267211	3.6205487	3.7719352	P	P	P
hsa-miR-5581-5p	0.000257219	5.124140975	4.11901	4.0121217	4.468674	P	P	P	1.9464353	1.577412	2.0079992	P	P	P
hsa-miR-5703	0.003932709	9.385621437	7.6519704	6.878999	6.693791	P	P	P	4.057878	3.6854112	3.9523053	P	P	P
hsa-miR-5739	0.000828804	3.03721306	8.073952	7.9665885	7.9665885	P	P	P	6.243199	6.400667	6.5422354	P	P	P
hsa-miR-574-3p	0.000275332	0.008675253	-3.1578648	-3.1905131	-3.1796591	A	A	A	3.6854112	3.4482384	3.8562999	P	P	P
hsa-miR-574-5p	0.002861744	2.621667892	7.908693	7.499739	7.61356	P	P	P	6.3576484	6.187962	6.330645	P	P	P
hsa-miR-575	0.000353482	5.582829197	6.3576484	6.400667	6.400667	P	P	P	3.7962844	4.0121217	3.8999321	P	P	P
hsa-miR-5787	0.006782815	5.368755202	8.795037	8.727931	8.654416	P	P	P	6.053328	6.029608	6.714629	P	P	P
hsa-miR-582-5p	0.000616761	0.001055617	-3.1578648	-3.1905131	-3.1796591	A	A	A	7.0410886	6.203362	6.7691016	P	P	P
hsa-miR-584-5p	0.000887239	0.002528786	-3.1578648	-3.1905131	-3.1796591	A	A	A	5.7690125	5.54527	4.9087005	P	P	P
hsa-miR-590-5p	0.000617427	0.233045639	3.8130388	4.236219	3.8267286	P	P	P	6.2206006	5.932713	6.053328	P	P	P
hsa-miR-6073	4.78E-05	0.00394821	-3.1578648	-3.1905131	-3.1796591	A	A	A	4.7664566	4.9331126	4.717377	P	P	P
hsa-miR-6085	0.001465397	3.875937515	7.88882	7.88882	7.6519704	P	P	P	5.54527	6.053328	5.932713	P	P	P
hsa-miR-6086	0.012760513	3.427147438	4.072406	3.7543721	3.9988286	P	P	P	2.6217492	1.713976	2.0270984	P	P	P
hsa-miR-6087	0.000394609	4.675252243	10.0384865	9.87587	10.348797	P	P	P	7.61356	8.1229725	7.846886	P	P	P
hsa-miR-6089	0.010666982	2.389711946	11.187136	10.529345	10.919219	P	P	P	9.521075	9.830814	9.568089	P	P	P
hsa-miR-6124	0.004068914	2.049207952	6.400667	6.3576484	6.273411	P	P	P	5.282623	5.1446013	5.4821186	P	P	P
hsa-miR-6126	0.008964552	19.28393103	4.1632457	4.085778	4.7004194	P	P	P	-1.2086849	0.40997612	0.48188725	P	P	P
hsa-miR-6127	0.002449305	2.242078642	8.576817	8.248948	8.373192	P	P	P	7.2188106	7.182649	7.3186646	P	P	P
hsa-miR-6129	0.003924523	27.65573126	4.7766256	4.4806085	3.8363023	P	P	P	0.44351065	-0.80410147	-1.3875048	P	P	P
hsa-miR-6131	0.01434223	2.761161105	5.093093	4.729522	5.3682666	P	P	P	3.6636858	3.6335928	3.5662074	P	P	P

hsa-miR-6132	0.001459051	4.936842559	6.714629	6.7691016	6.714629	P	P	P	4.3566866	4.6215453	4.288173	P	P	P
hsa-miR-622	0.001586373	119.4108808	3.935175	3.010559	3.6479452	P	P	P	-3.3194392	-3.3219283	-3.3162193	A	A	A
hsa-miR-625-5p	0.000441172	3.98094227	7.297641	7.0410886	7.079769	P	P	P	5.334731	4.9087005	5.177499	P	P	P
hsa-miR-628-5p	0.000778328	0.005235037	-3.1578648	-3.1905131	-3.1796591	A	A	A	4.27377	4.057878	4.7766256	P	P	P
hsa-miR-630	0.001905508	10.26902475	8.1229725	7.576128	7.3186646	P	P	P	4.510607	4.186671	4.3413744	P	P	P
hsa-miR-642a-3p	0.008365989	4.76016756	8.97648	9.154404	9.976183	P	P	P	6.878999	7.244012	7.3909016	P	P	P
hsa-miR-642b-3p	0.000556435	3.880914596	6.053328	6.147969	6.5153904	P	P	P	4.11901	4.260568	4.4862566	P	P	P
hsa-miR-6510-5p	0.002613138	2.673367053	6.187962	5.9008718	5.8190274	P	P	P	4.536281	4.27377	4.817203	P	P	P
hsa-miR-652-3p	0.000370482	0.093129819	3.6335928	3.232212	2.911333	P	P	P	6.5422354	6.978785	6.579298	P	P	P
hsa-miR-652-5p	0.000103607	19.82418225	3.5811896	2.9259632	3.139044	P	P	P	-1.3053184	-0.6937595	-1.2912214	P	P	P
hsa-miR-660-5p	6.39E-05	0.23432579	3.0562327	2.8766868	2.8519025	P	P	P	5.0731435	4.952126	5.0457525	P	P	P
hsa-miR-664b-5p	0.003371247	0.293289229	4.468674	3.875716	3.7962844	P	P	P	6.098819	5.831571	5.5676794	P	P	P
hsa-miR-6717-5p	0.000886136	2.643728076	5.1446013	4.826829	5.093093	P	P	P	3.605049	3.5375905	3.7274342	P	P	P
hsa-miR-718	0.000194296	23.56444866	2.947276	3.0973506	3.201806	P	P	P	-1.4622538	-1.495435	-1.4604862	P	P	P
hsa-miR-762	0.003376058	2.910192317	5.9179406	5.932713	6.4438534	P	P	P	4.400464	4.544902	4.7664566	P	P	P
hsa-miR-940	0.012651316	2.924519917	4.7664566	5.200648	5.402697	P	P	P	3.5811896	3.5490105	3.6636858	P	P	P
hsa-miR-98-5p	0.001448969	0.41040903	5.795355	5.9585047	5.734473	P	P	P	7.135916	7.079769	7.135916	P	P	P
hsa-miR-99b-5p	0.00207907	0.005534124	-3.1578648	-3.1905131	-3.1796591	A	A	A	4.4602914	3.5811896	4.7004194	P	P	P
